# Comprehensive Adsorption–Desorption Kinetics of Shaped UTSA-16(Co)/Polyacrylate Composites for Fixed-Bed CO_2_ Capture

**DOI:** 10.3390/nano16211341

**Published:** 2026-10-22

**Authors:** Xiangmei Zhang, Chunlai Wang, Zhejiaji Zhu, Wanyue Zang, Yuan Sun, Li Lv

**Affiliations:** 1 State Key Laboratory of Chemistry for NBC Hazards Protection, Beijing 100191, China; 2 College of Chemical Engineering, Beijing University of Chemical Technology, Beijing 100029, China; 3 Tianjin Key Laboratory of Green Chemical Technology and Process Engineering, Tiangong University, Tianjin 300387, China

**Keywords:** CO_2_ capture, UTSA-16(Co), MOF–polymer composite, stepwise impregnation, fixed-bed adsorption, adsorption kinetics

## Abstract

Pristine metal–organic frameworks (MOFs) generally suffer from low mechanical strength and poor processability, hindering their direct application in fixed-bed adsorption systems. In this work, we propose a stepwise impregnation strategy that enables uniform in-situ crystallization of UTSA-16(Co) within amino-functionalized polyacrylate (PAA-NH_2_) matrices via pre-anchored cobalt ion sites, designed to overcome the low loading and inhomogeneous distribution issues of conventional one-step impregnation methods. Three synthesis protocols (one-step impregnation, stepwise impregnation, and secondary growth) were systematically compared, and the stepwise approach achieved the highest MOF loading of 53 wt%. The optimal composite exhibited a thermogravimetric (TGA) pure-CO_2_ uptake of 95.0 mg/g at 25 °C and 1 bar and retained 98.6% of its initial capacity after 10 consecutive adsorption–desorption cycles, indicating good short-term cyclic stability. Systematic fixed-bed tests revealed that the empirical Avrami fractional-order model (R^2^ ≥ 0.994) accurately described the heterogeneous adsorption kinetics of the composite, and the dynamic breakthrough behavior was jointly regulated by gas flow rate, bed temperature, inlet CO_2_ concentration, and relative humidity. The composite was found to be moisture-sensitive: at 60% RH, the capacity fell by 52.6%. A desorption efficiency above 99% was achieved within 20 min under optimal conditions (90 °C, 0.6 L/min purge gas). Overall, this work provides systematic kinetic data and operational guidelines for the optimization of MOF–polymer composite adsorbents in laboratory-scale fixed-bed applications.

## 1. Introduction

Carbon capture and storage (CCS) has been widely recognized as a key global strategy for mitigating carbon emissions [1]. Among various carbon capture technologies, adsorption-based separation has drawn extensive attention due to its flexible operation, low energy consumption, and strong adaptability to diverse working conditions. Accordingly, the development of high-performance adsorbents is critical to the practical application of this technology [2,3]. As a class of high-performance adsorbents, metal–organic frameworks (MOFs) feature ultrahigh specific surface areas, highly tunable pore structures, and designable active sites and are widely regarded as one of the most promising next-generation CO_2_ adsorbents [4,5]. However, pristine MOFs are generally synthesized in fine powder form, and their direct packing into fixed-bed reactors often brings severe engineering challenges, including excessive pressure drop across the fixed bed, non-uniform gas flow distribution, and adsorbent entrainment, which greatly restrict their large-scale industrial application [6]. For comparison, the traditional CO_2_ capture routes and adsorbents each have distinct limitations. Aqueous amine scrubbing (e.g., monoethanolamine) offers high selectivity and capacity but suffers from a large regeneration energy penalty, solvent volatility, and equipment corrosion. Conventional solid adsorbents include zeolites (e.g., 13X), which display high dry CO_2_ capacity but are strongly hydrophilic and readily poisoned by water, requiring high regeneration temperatures; activated carbons, which are water-tolerant and cheap but have low CO_2_/N_2_ selectivity and weak low-partial-pressure uptake; and amine-grafted silicas, which improve selectivity but can suffer from amine leaching and oxidative degradation. In comparison, MOFs such as UTSA-16 combine high pore volume, open Co^2+^ sites, and a high volumetric capacity at near-ambient conditions [7], while retaining better structural tunability; their principal drawback for deployment is the powder-form shaping problem noted above. This motivates the development of a shaped MOF composite that preserves the intrinsic capacity of the MOF while adding macroscopically processable geometry.

A feasible strategy to solve the above issues is to confine MOF growth within shaped porous matrices. For instance, Gaikwad et al. [8] fabricated self-standing PAN/UTSA-16 composite fibers via electrospinning, which retained over 75% of the CO_2_ adsorption capacity of the pristine MOF and exhibited excellent mechanical stability, preliminarily verifying the engineering feasibility of MOF shaping strategies. Other structured MOF/polymer systems include UTSA-16 grown in 3D-printed kaolin monoliths [9], NbOFFIVE-1-Ni embedded in polyacrylate for direct air capture [10], and SIFSIX-3-Ni or UiO-66/ZIF-8 shaped into PAN beads [11,12]. Most of these composites are, however, obtained by physically mixing pre-synthesized MOF powder with a binder or by one-pot co-infiltration, which readily causes pore blockage and MOF aggregation, creating a trade-off between loading and dispersion. Furthermore, ideal adsorbents for industrial scenarios need to meet comprehensive performance requirements beyond high static adsorption capacity, such as fast adsorption kinetics, low desorption energy consumption, and long-term cyclic stability. Hence, mechanical feasibility alone cannot guarantee industrial applicability. Although Zhang et al. [13] reported the promising breakthrough performance of ZU-16-Co in low-pressure mixed gas systems, most existing studies still focus on static adsorption capacity, while systematic quantitative investigations on the multi-variable coupling mechanism and desorption behavior under dynamic operating conditions remain limited. As a result, conclusions from existing studies cannot provide direct guidance for the engineering scale-up and process design of fixed-bed systems [14].

Against this background, the present work is distinguished from the shaping routes above by three aspects of novelty. (i) Rather than loading a pre-synthesized MOF, this route first pre-anchors the cobalt precursor onto the amino sites of a porous PAA bead and only then is the citrate ligand introduced, which decouples metal loading from ligand-induced bulk precipitation and drives spatially uniform, nanoscale nucleation (100–200 nm) inside the pore channels. (ii) Three loading routes (one-step, stepwise, and secondary growth) are compared head-to-head on the same matrix, linking preparation route directly to Co content, MOF loading, surface area, pore volume, and capacity. (iii) Beyond static capacity, the optimal composite is evaluated across the full set of dynamic fixed-bed variables (flow rate, temperature, inlet concentration, humidity, and bed height) together with thermal purge desorption, and the kinetic data are analyzed with empirical models to provide operationally useful, quantitative guidelines.

To address this research gap, this work developed a stepwise impregnation strategy, in which metal salts and organic ligands were sequentially introduced into amino-functionalized polyacrylate (PAA-NH_2_) matrices to realize the in-situ crystallization of UTSA-16(Co) within the pore channels. First, three preparation routes (one-step impregnation, stepwise impregnation, and secondary growth) were compared in terms of microstructure and adsorption performance. After the optimal sample was determined, its dynamic performance was evaluated in a fixed-bed system to investigate the effects of gas flow rate, bed temperature, inlet CO_2_ concentration, relative humidity (RH), and bed height on breakthrough characteristics. Finally, the Avrami fractional-order kinetic model was applied to empirically describe the mass transfer kinetics, and key parameters for the desorption process were optimized. This work provides systematic kinetic data and design guidelines for the laboratory-scale application of MOF-based composite adsorbents in fixed-bed systems.

## 2. Materials and Methods

### 2.1. Materials

Glycidyl methacrylate (GMA, 85.0%), trimethylolpropane triacrylate (TMPTA, ≥99.0%), N,N,N′,N′-tetramethylethylenediamine (TMEDA, 99%), N,N-dimethylaniline (DMA, 98%), cobalt acetate tetrahydrate (Co(CH_3_COO)_2_·4H_2_O, 99.5%), zinc acetate dihydrate (Zn(CH_3_COO)_2_·2H_2_O, 98%), potassium hydroxide (KOH, 90%), tetraethylenepentamine (TEPA, 95%), polyethyleneimine (PEI, Mw = 600, 99%), and polyethyleneimine (PEI, Mw = 1800, 99%) were purchased from Shanghai Macklin Biochemical Co., Ltd. (Shanghai, China). Toluene (C_6_H_5_CH_3_, ≥99.5%) was purchased from Sinopharm Chemical Reagent Co., Ltd. (Shanghai, China). Polyvinyl alcohol 1788 (PVA, 87.0%), ammonium persulfate (APS, 98.5%), benzoyl peroxide (BPO, ≥99%), citric acid monohydrate (C_6_H_8_O_7_·H_2_O, 99.5%), methanol (99.5%), and ethanol (99.7%) were purchased from Shanghai Aladdin Biochemical Technology Co., Ltd. (Shanghai, China). The N_2_ (99.99%) and CO_2_ (99.9%) gases were obtained from Beijing Haipu Gas Co., Ltd. ( Beijing, China). All the chemicals were commercially available and employed without additional purification. The deionized water used was from a local source.

### 2.2. Amino Functionalization of Polyacrylate Substrates

Spherical polyacrylate (PAA) matrices were synthesized via emulsion suspension polymerization according to a previously reported procedure [15], followed by amino functionalization via an impregnation route [16]. Briefly, 0.4 g of dried PAA matrix was placed in a 25 mL round-bottom flask and evacuated for 30 min to fully remove trapped air from the pore channels. A mixed solution of PEI 1800 and methanol (4:1 mass ratio) was prepared, sonicated to achieve complete dissolution, and injected into the flask under vacuum to infiltrate the solution into the pore channels of the matrix. The flask was then heated at 130 °C for 120 min under a N_2_ atmosphere. The resulting product was washed alternately with deionized water and methanol until the filtrate reached neutral pH and then vacuum-dried at 70 °C for 120 min to yield PAA-NH_2_.

### 2.3. Synthesis of UTSA-16(Co)/Polyacrylate Composites

The as-prepared PAA-NH_2_ was used as the polymer matrix for the in-situ crystallization of UTSA-16(Co) via three distinct protocols: one-step impregnation, stepwise impregnation, and secondary growth.

(1) One-Step Impregnation Method

A precursor solution containing both the metal salt and organic ligand was prepared. Specifically, 1.2450 g of Co(CH_3_COO)_2_·4H_2_O was dissolved in 12.5 mL of deionized water (Solution A), and 1.0500 g of C_6_H_8_O_7_·H_2_O together with 0.8400 g of KOH was dissolved in 12.5 mL of ethanol (Solution B). Solutions A and B were then mixed and sonicated for 15 min. Separately, 0.2 g of PAA-NH_2_ was evacuated in a 50 mL flask for 30 min, after which the mixed precursor solution was introduced, and the system was maintained under vacuum for 10 min to impregnate the matrix. The mixture was subsequently transferred to a Teflon-lined autoclave and reacted at 120 °C for 48 h. The product was washed with methanol and vacuum-dried at 90 °C for 12 h, yielding UTSA-16(Co)/PAA-O.

(2) Stepwise Impregnation Method

A stepwise impregnation strategy was adopted to mitigate the non-uniformity of MOF loading caused by solubility differences between the components. First, 0.2 g of PAA-NH_2_ was pre-evacuated for 30 min, immersed in an aqueous cobalt acetate solution (1.2450 g in 12.5 mL of water) for 10 min, and then dried at 90 °C for 120 min to immobilize cobalt ions on the inner pore surfaces. After a second evacuation step, the citric acid–KOH ethanol solution (1.0500 g of C_6_H_8_O_7_·H_2_O and 0.8400 g of KOH in 12.5 mL of ethanol) was introduced, and the system was kept under vacuum for another 10 min. The subsequent hydrothermal reaction, washing, and drying procedures were identical to those of the one-step impregnation method. The final product was denoted as UTSA-16(Co)/PAA-S.

(3) Secondary Growth Method

UTSA-16(Co)/PAA-S was used as the matrix, and the stepwise impregnation–hydrothermal crystallization procedure was repeated once to enhance the MOF loading and crystallinity. After the same washing and drying treatments, the resulting sample was designated as UTSA-16(Co)/PAA-T.

### 2.4. Characterization

The physicochemical properties of the as-synthesized composites were systematically investigated. First, the crystalline phase was determined via X-ray diffraction (XRD) on a Rigaku SmartLab SE diffractometer with Cu Kα radiation (λ = 1.5406 Å), in the 2θ range of 5–50° at a scanning rate of 2°/min. Subsequently, the surface functional groups were characterized via Fourier-transform infrared (FTIR) spectroscopy on a Thermo Scientific Nicolet iS20 spectrometer, using the KBr pellet technique in the wavenumber range of 4000–400 cm^−1^ with a resolution of 4 cm^−1^. Furthermore, the micromorphology and elemental distribution were observed via a field-emission scanning electron microscope (FESEM, ZEISS Gemini 300, Oberkochen, Germany) coupled with an energy-dispersive X-ray spectroscopy (EDS) detector for elemental mapping. Additionally, N_2_ adsorption–desorption isotherms were tested at −196 °C on a Micromeritics ASAP 2460 analyzer, after vacuum degassing at 90 °C for 5 h. The specific surface area and micropore volume were derived from the isotherms via the Brunauer–Emmett–Teller (BET) method [17] and the t-plot approach, respectively, and the average pore diameter was obtained by the DFT/BJH method, as summarized in Table S1. Finally, the thermal stability was evaluated via thermogravimetric analysis (TGA) on a Netzsch 209 F3 analyzer, from room temperature to 700 °C at a heating rate of 10 °C/min under a N_2_ flow of 20 mL/min.

### 2.5. Adsorption Performance Tests

(1) CO_2_ Isothermal Adsorption Tests

Static CO_2_ adsorption isotherms of the as-prepared samples were collected at 0 °C and 25 °C over 0–1 bar using a Quantachrome Autosorb IQ gas adsorption analyzer. Before each test, approximately 100 mg of the sample was degassed at 90 °C for 12 h. These static isotherms were used to confirm the type-I, predominantly physisorptive character of the uptake; the reported pure-CO_2_ capacities were obtained gravimetrically by TGA. Equilibrium adsorption capacities at different partial pressures were derived, and the obtained isotherm data were fitted using the Langmuir [18] and Freundlich [19] models (Equations (S1) and (S2)).

(2) Thermogravimetric Adsorption and Cyclic Stability Tests

To evaluate the gravimetric adsorption capacity under pure CO_2_ and the cyclic stability of the samples, CO_2_ adsorption–desorption cycle tests were performed on a Netzsch 209 F3 analyzer. Approximately 15 mg of each sample was first activated at 120 °C under a N_2_ atmosphere (20 mL/min) for 60 min. After the sample was cooled to the preset adsorption temperature, the gas atmosphere was switched to pure CO_2_ for 60 min to allow adsorption, while the sample mass was recorded in real time. For desorption, the sample was heated to the preset desorption temperature at a rate of 10 °C/min under N_2_ flow, and then held isothermally for 60 min. This adsorption–desorption cycle was repeated 10 times to assess the short-term cyclic stability. The pure-CO_2_ gravimetric uptake was obtained from this test, not from the static volumetric isotherm. The adsorption capacity was calculated using Equation (1):

(1) $$q_{t}=\frac{m_{t}-m_{0}}{m_{0}}\times 1000$$

where ***t*** is the CO_2_ adsorption time (min), ***q_t_*** is the CO_2_ adsorption capacity at time t (mg/g), ***m_t_*** is the total mass of the sample at time t (g), and ***m*_0_** is the initial mass of the activated sample (g).

(3) Adsorption–Desorption Kinetics Tests

Dynamic adsorption–desorption experiments were carried out in a fixed-bed system under atmospheric pressure to evaluate the breakthrough characteristics and desorption performance under continuous flow conditions. The experimental setup consisted of a stainless-steel fixed-bed reactor (inner diameter: 20 mm) equipped with gas mixing, thermostatic adsorption control, and online gas detection modules. Compressed air and CO_2_ gas streams were controlled by mass flow controllers, and a bubbling-type humidifier was used for humidity regulation. For humid tests, the relative humidity was established by mixing the dry feed with a water-saturated stream generated by the bubbling humidifier and was monitored in real time by an inline hygrometer located at the bed inlet; the bed was equilibrated until the inlet RH remained stable within ±2% for at least 15 min before the CO_2_ feed was introduced, whereas the dry baseline corresponded to an RH below 1%. A three-way solenoid valve enabled rapid switching between the test line and bypass line, and a non-dispersive infrared (NDIR) analyzer was used to continuously monitor the outlet CO_2_ concentration.

In the adsorption breakthrough tests, a precisely weighed adsorbent sample (approximately 1.5 g for a 1 cm bed height) was loaded into the fixed-bed column and packed by gentle tapping to the target bed height, with quartz wool placed at both ends to retain the particles, and activated under a dry air atmosphere at the preset activation temperature for 180 min. After the fixed bed was cooled to the target temperature, a CO_2_/air mixed gas was introduced into the bed. The outlet CO_2_ concentration C_t_ was recorded continuously. Breakthrough was defined as C_t_/C_0_ = 0.05, and adsorption saturation was deemed to be achieved when C_t_/C_0_ reached 1.0 and remained stable for 10 min. Breakthrough curves were plotted with C_t_/C_0_ on the *y*-axis and time t on the *x*-axis, and the dynamic saturated adsorption capacity was calculated via numerical integration using Equation (S3) [20]. To further reveal the CO_2_ adsorption mechanism of UTSA-16(Co)/PAA-S, the dynamic adsorption data were fitted with the empirical pseudo-first-order, pseudo-second-order, and Avrami kinetic models (Equations (S4)–(S6)) [21].

In the desorption experiments, the sample (bed height: 1 cm) was first saturated at 25 °C with 1 vol% CO_2_ at a flow rate of 0.3 L/min. The CO_2_ feed was then cut off, and CO_2_-free dry, purified air at the preset temperature and flow rate was introduced to purge the bed for 60 min, during which the outlet CO_2_ concentration was recorded continuously. The CO_2_ desorption capacity per unit mass of adsorbent was calculated by integrating the desorption curve (Equation (S7)), and the desorption efficiency was obtained using Equation (S8). Each fixed-bed adsorption and desorption condition was determined in a single experimental run, and the breakthrough and desorption curves are the corresponding individual, un-averaged data. Likewise, the TGA uptake and the 10-cycle results are single determinations. All capacities were calculated from these individual curves.

## 3. Results and Discussion

### 3.1. Morphological and Structural Characterization

**Figure 1:**
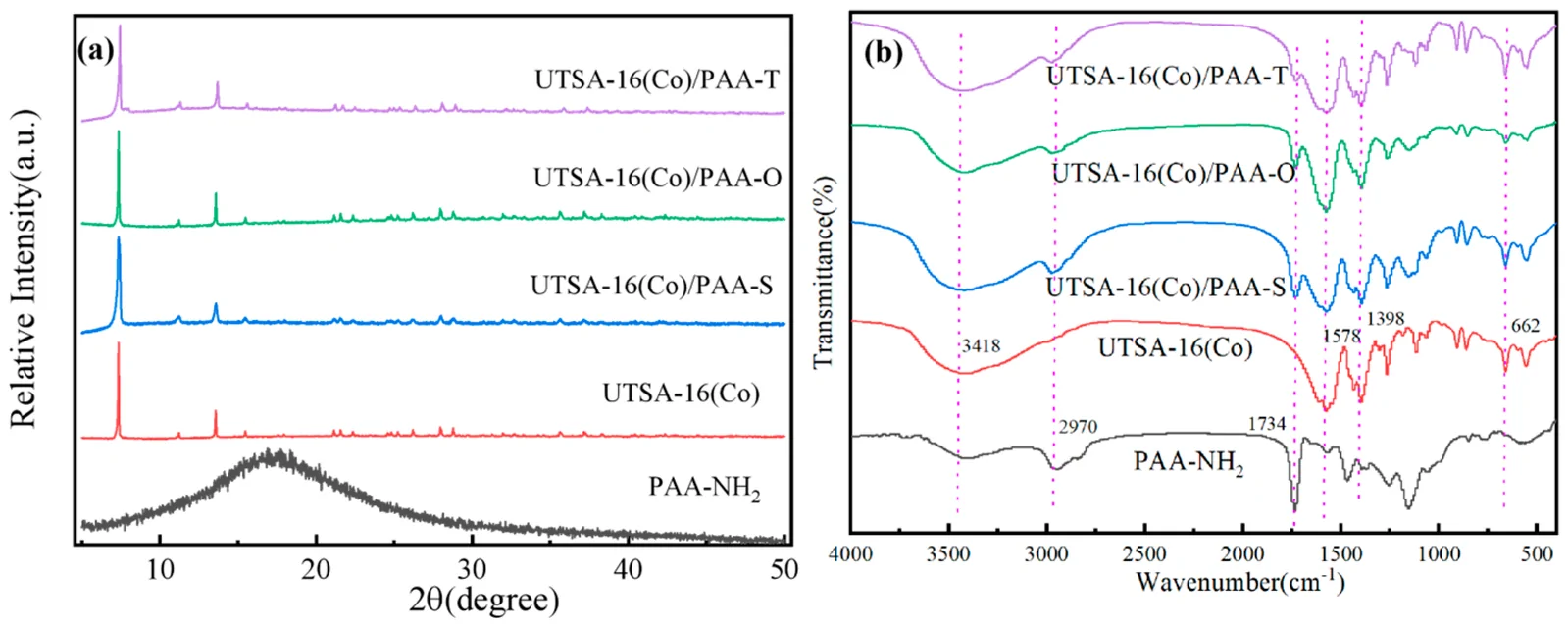
(**a**) XRD patterns and (**b**) FTIR spectra of PAA-NH_2_, UTSA-16(Co), UTSA-16(Co)/PAA-O, UTSA-16(Co)/PAA-S and UTSA-16(Co)/PAA-T.

The successful immobilization of UTSA-16(Co) within the PAA-NH_2_ matrix was confirmed by XRD and FTIR characterization. Figure 1a shows the XRD patterns of the three composites. All samples displayed sharp, well-defined characteristic diffraction peaks at 11.3°, 13.8°, 21.3°, and 28.4°, matching those of pristine UTSA-16(Co). These results confirmed that MOF crystallites were successfully immobilized within the PAA-NH_2_ matrix and maintained their highly ordered crystalline structure after the hydrothermal growth and impregnation processes, with no detectable crystallinity deterioration. The pristine PAA-NH_2_ support showed no sharp reflections but only a broad diffuse halo centered at approximately 18–20°, which was the characteristic amorphous scattering band of the cross-linked polyacrylate network and indicated that the polymer support itself was essentially non-crystalline; this band was largely masked in the composites once the strongly scattering MOF phase was introduced.

FTIR spectra (Figure 1b) further confirmed the successful integration of the MOF phase into the polymeric matrix. Specifically, all three composites exhibited the characteristic absorption bands of the polyacrylate matrix. The peak at 1734 cm^−1^ was assigned to the C=O stretching vibration of acrylate moieties, while the band at 2970 cm^−1^ corresponded to the asymmetric stretching vibration of –CH_2_ groups [22]. The broad band at 3418 cm^−1^ was attributed to overlapping N–H stretching of the grafted amino groups and O–H stretching of adsorbed water, supporting the presence of amine anchoring sites; the bands near 1450 cm^−1^ and in the 1250–1050 cm^−1^ region were assigned to –CH_2_– scissoring and to C–N/C–O stretching of the amine and ester groups, respectively. In addition, the bands at 1578 cm^−1^ and 1398 cm^−1^ were attributed to the asymmetric and symmetric stretching vibrations of carboxylate groups formed upon citrate coordination, respectively. The characteristic peak at 662 cm^−1^ was assigned to the Co–O stretching vibration in the UTSA-16(Co) framework. The simultaneous presence of these characteristic peaks originating from both the matrix and the MOF phase, together with the retained N–H bands, was consistent with the in-situ growth of UTSA-16(Co) within the internal pore channels of the PAA-NH_2_ matrix. Direct spectroscopic evidence for Co–amine coordination (such as XPS) is not yet available, so this pre-anchored Co–amine pathway is presented as a working hypothesis rather than a demonstrated mechanism.

**Figure 2:**
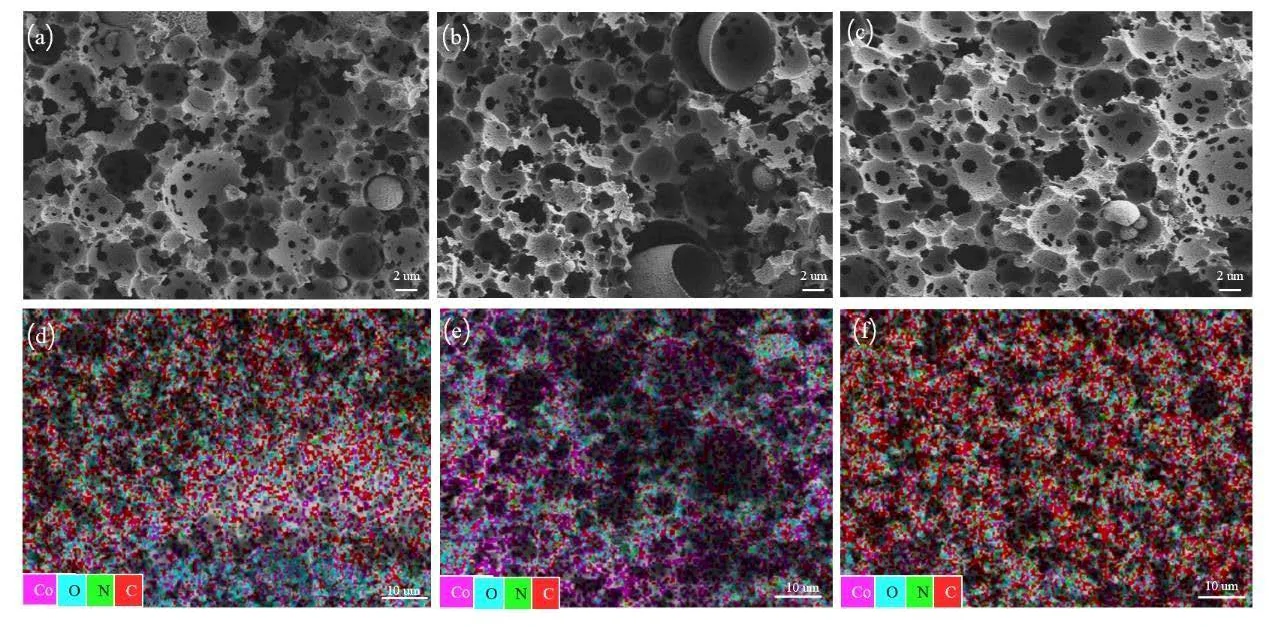
SEM images and EDS elemental mappings of (**a**,**d**) UTSA-16(Co)/PAA-O, (**b**,**e**) UTSA-16(Co)/PAA-S and (**c**,**f**) UTSA-16(Co)/PAA-T.

SEM and EDS mapping results (Figure 2) revealed the loading morphology of MOF crystallites and the spatial distribution of elements within the pore channels of the matrix. For the UTSA-16(Co)/PAA-S prepared via stepwise impregnation, MOF crystallites were uniformly and densely dispersed throughout the interior pore channels of the microspheres. Compared with pristine MOF crystals, the crystallites grown in the confined pore channels exhibited a smaller particle size of approximately 100–200 nm, corresponding to a size reduction of over 50% relative to the pristine crystals. In comparison, the sample prepared via one-step impregnation exhibited lower homogeneity of MOF distribution, whereas the secondary growth method yielded the lowest loading efficiency, with only sparse MOF crystallites scattered throughout the microspheres. This poor loading performance was attributed to the structural disruption of both pre-formed MOF crystallites and the matrix pore structure during secondary crystallization.

EDS elemental mapping revealed the spatial distribution of Co (marked in purple) across the cross-sections of the composite microspheres. The EDS spectra acquired together with the elemental mappings in Figure 2 contained only the expected Co, C, O, and N signals with no detectable impurity peaks, confirming phase purity. Quantitative analysis showed that the Co mass fractions in the cross-sectional areas of UTSA-16(Co)/PAA-S, UTSA-16(Co)/PAA-O, and UTSA-16(Co)/PAA-T were 13.28%, 8.13%, and 7.5%, respectively, which were in good agreement with the SEM observations. These results demonstrated that the stepwise impregnation strategy was more conducive to the uniform nucleation and growth of MOF crystals within the confined pore space of the polymer matrix, thus enabling a higher loading of active components.

**Figure 3:**
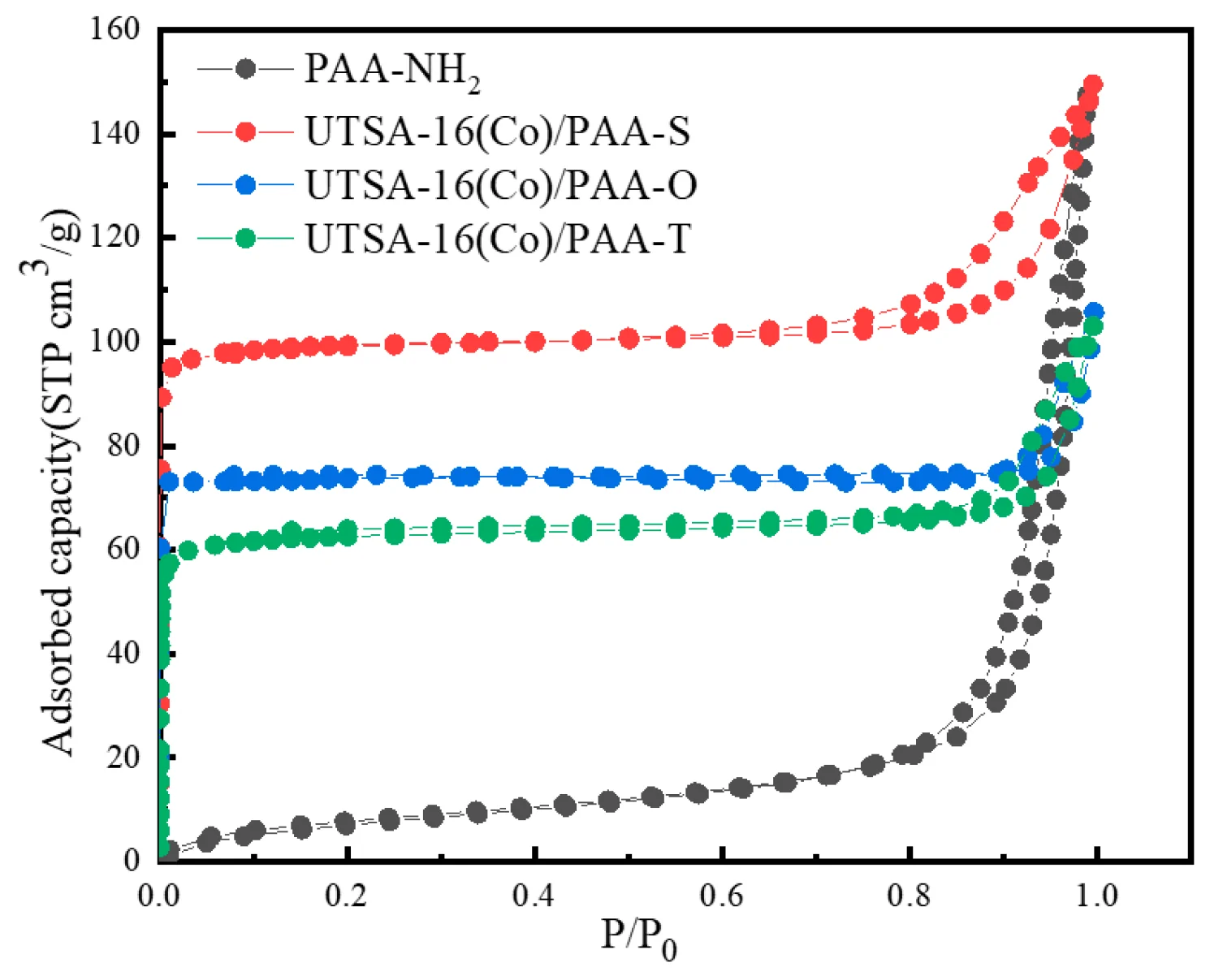
N_2_ adsorption–desorption isotherms of PAA-NH_2_, UTSA-16(Co)/PAA-O, UTSA-16(Co)/PAA-S and UTSA-16(Co)/PAA-T.

The pore structures of the composites were further characterized via N_2_ adsorption–desorption measurements (Figure 3). Pristine PAA-NH_2_ exhibited a typical type IV isotherm with a specific surface area of 35.26 m^2^/g, consistent with the presence of mesopores and inter-particle voids in its polymeric framework. In contrast, all composites exhibited hybrid isotherms with combined type I and type IV features. At very low relative pressures (P/P_0_ < 0.05), the sharp rise in N_2_ uptake corresponded to micropore filling within the UTSA-16(Co) framework, while the pronounced increase in adsorption capacity at P/P_0_ = 0.8–0.95 indicated mesoporous transport channels and inter-particle voids within the packed polymer beads, complementing the MOF micropores to form a hierarchical micro-/mesoporous network. Furthermore, Table S1 shows that the average pore sizes of the three composites were all larger than that of pristine UTSA-16(Co), owing to the inherently large average pore size (26.42 nm) of the PAA-NH_2_ matrix. For UTSA-16(Co)/PAA-S, the micropore-filling step was shifted to slightly lower P/P_0_ and was more pronounced than for the other composites; this indicated a larger fraction of narrow, well-developed micropores introduced by the higher, more uniform MOF loading (i.e., greater micropore volume and stronger low-pressure adsorption), rather than a loss of porosity. Notably, UTSA-16(Co)/PAA-S exhibited the highest specific surface area (407.46 m^2^/g) and micropore volume (0.1448 cm^3^/g), consistent with its highest MOF loading. Consequently, this hierarchical micro-/mesoporous structure not only retained abundant microporous adsorption sites from the MOF phase but also reduced gas mass transfer resistance through the mesoporous transport channels and inter-particle voids of the polymer matrix, thus providing a favorable structural foundation for rapid molecular diffusion in subsequent dynamic adsorption processes.

**Figure 4:**
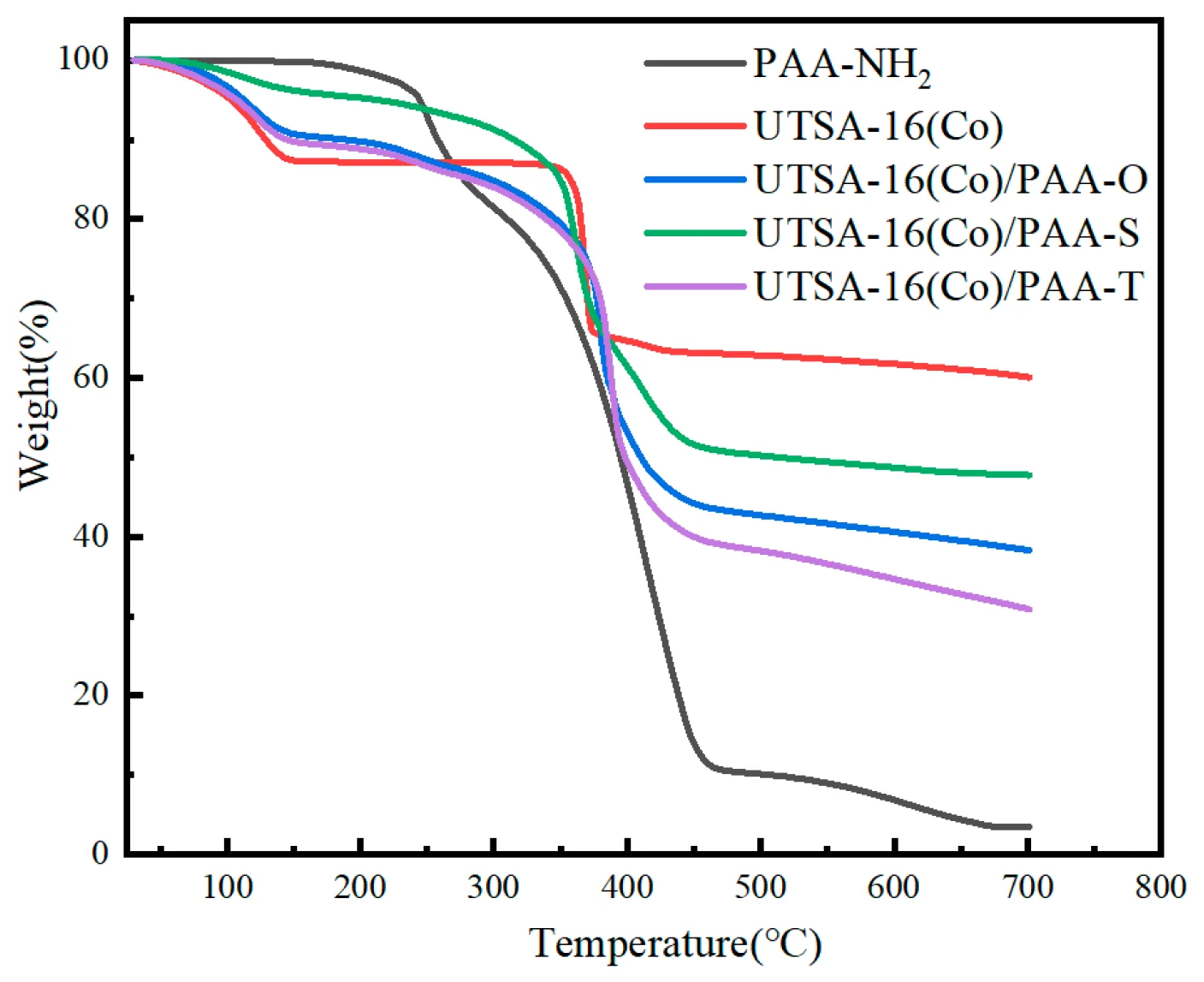
TGA curves of UTSA-16(Co), PAA-NH_2_, UTSA-16(Co)/PAA-O, UTSA-16(Co)/PAA-S and UTSA-16(Co)/PAA-T.

The thermal stability of the composites was further evaluated via TGA measurements (Figure 4). Pristine PAA-NH_2_ exhibited two distinct thermal degradation stages. An initial mass loss of approximately 5% occurred around 180 °C, which was attributed to the removal of residual solvents and adsorbed moisture. This was followed by a pronounced decomposition stage above 238 °C, with a total mass loss exceeding 90% at 475 °C, corresponding to complete degradation of the polymer backbone. In contrast, the thermogravimetric profiles of the three composites closely resembled those of pristine UTSA-16(Co), with rapid thermal decomposition occurring above 300 °C. The final total mass losses were 62% for UTSA-16(Co)/PAA-O, 51% for UTSA-16(Co)/PAA-S, and 70% for UTSA-16(Co)/PAA-T. Among these, UTSA-16(Co)/PAA-S exhibited the lowest mass loss, which was directly related to its highest MOF loading. Notably, in the low-temperature range from room temperature to 250 °C, the composites exhibited less thermal mass loss than pristine UTSA-16(Co) powder, indicating that the encapsulation and confinement effects of the PAA-NH_2_ matrix moderately enhanced the thermal stability of the composite system, whose degradation onset (above 300 °C) was well above the operating window used here (25–110 °C) and thus ensured stable operation under the present laboratory conditions. The above microstructural characterization results confirmed that the stepwise impregnation route endowed the composite with superior MOF loading, dispersion uniformity, porosity, and thermal stability.

### 3.2. Effect of Organic Amine Species on Adsorption Performance

Since the epoxy groups on the polyacrylate surface could react with amino groups via ring-opening reactions, the molecular structures of the organic amines determined the density and spatial distribution of active sites on the polymer matrix and further modulated the in-situ growth behavior of UTSA-16(Co). To investigate this effect, PEI 600, PEI 1800, and TEPA were selected as modifying agents, yielding functionalized matrices denoted as PAA-P6, PAA-P18, and PAA-TEPA, respectively. MOF crystallites were subsequently grown in situ under identical stepwise impregnation and synthesis conditions to fabricate UTSA-16(Co)/PAA-S-P6, UTSA-16(Co)/PAA-S-P18, and UTSA-16(Co)/PAA-S-T. The MOF mass loading calculated from Equation (S9) was 47.0%, 53.0%, and 37.5% for the three samples, respectively. This variation in MOF loading was mainly attributed to differences in the molecular weight, molecular structure, and retention capacity of the amine molecules. PEI, with long macromolecular chains and high nitrogen content, was more readily retained within the three-dimensional pore network of the matrix, where it was expected to provide abundant coordination anchoring sites for MOF nucleation and growth. In contrast, TEPA with a relatively low molecular weight was prone to leaching during the impregnation and subsequent washing steps, resulting in fewer available active sites.

The CO_2_ adsorption performance of the three composites was evaluated at 30 °C under various flow rates (Figure S1, TGA method under pure CO_2_). UTSA-16(Co)/PAA-S-P6 achieved an adsorption capacity of 55.0 mg/g at 50 mL/min, which remained relatively stable over the entire tested flow rate range. UTSA-16(Co)/PAA-S-P18 showed a similar dependence on flow rate but much higher adsorption capacities, reaching a maximum of 84.9 mg/g at 75 mL/min, which was approximately 1.53 times that of the P6-derived sample. In contrast, the CO_2_ adsorption capacity of UTSA-16(Co)/PAA-S-T was barely affected by variations in CO_2_ flow rate. Within the tested flow rate range of 25–100 mL/min, the equilibrium adsorption capacities of the three samples consistently followed the order: P18 > P6 > TEPA. These results indicated that modification with PEI 1800 effectively optimized the pore chemical environment of the matrix and promoted the uniform nucleation and high-quality crystal growth of UTSA-16(Co), thereby endowing the composite with superior mass transfer properties and adsorption performance.

### 3.3. Effect of Adsorption Temperature on Adsorption Performance

With PAA-P18 as the optimal polymer matrix, the effect of adsorption temperature on the CO_2_ adsorption performance of the three composites (UTSA-16(Co)/PAA-O, UTSA-16(Co)/PAA-S, and UTSA-16(Co)/PAA-T) was investigated. The MOF mass loading calculated from Equation (S9) was 41%, 53%, and 32% for the three samples, respectively. Among the three samples, UTSA-16(Co)/PAA-T exhibited the lowest loading, consistent with the SEM observations. This lower loading was attributed to the repeated thermal cycles and solvent exchange steps involved in the secondary growth process, which caused irreversible damage to both pre-synthesized MOF crystallites and the inherent pore structure of the matrix.

**Figure 5:**
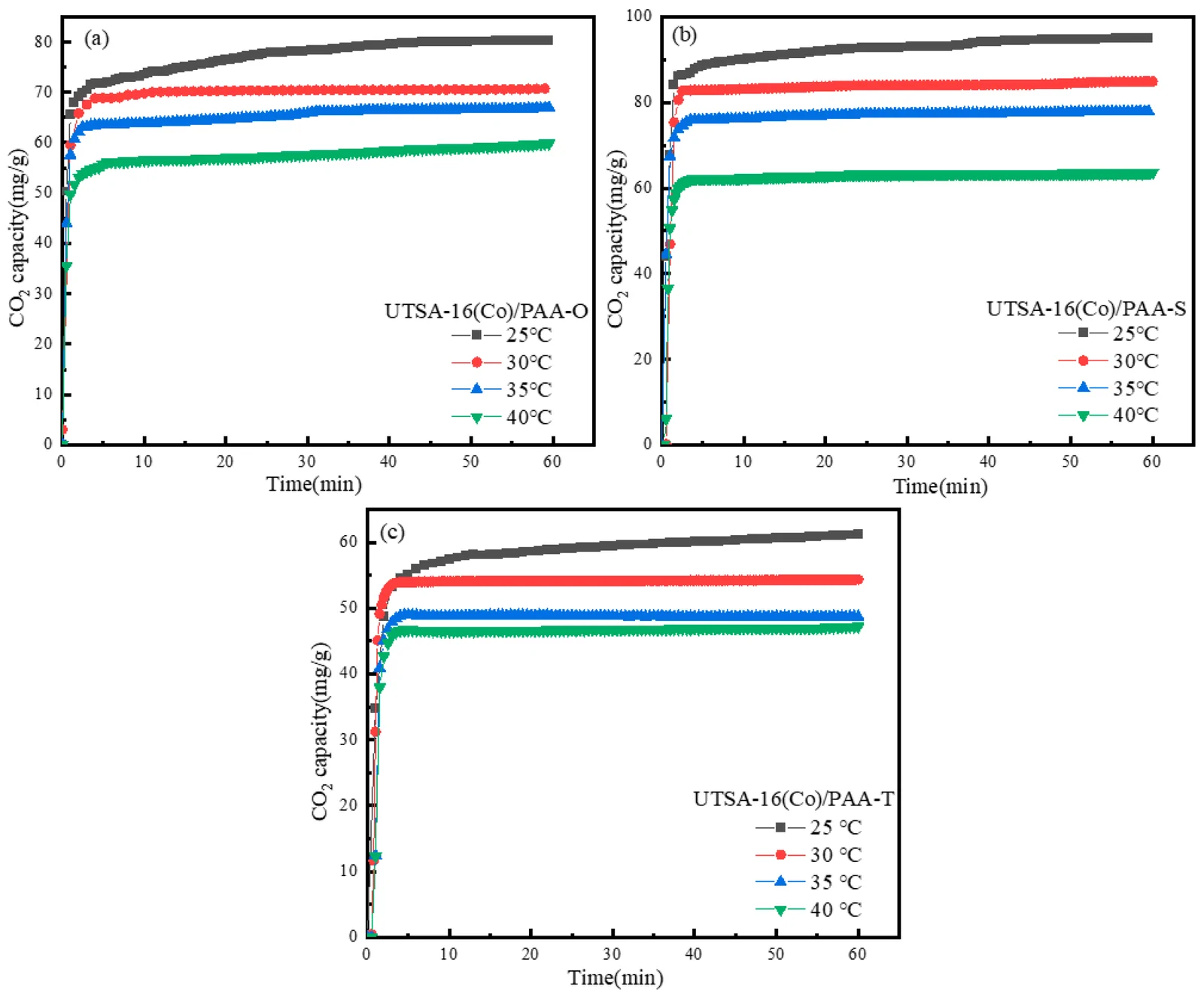
Adsorption curves of (**a**) UTSA-16(Co)/PAA-O, (**b**) UTSA-16(Co)/PAA-S and (**c**) UTSA-16(Co)/PAA-T at different adsorption temperatures.

Since CO_2_ capture over UTSA-16(Co) was dominated by physisorption, the adsorption temperature was a critical thermodynamic parameter governing the adsorption capacity. Figure 5 presents the pure-CO_2_ TGA adsorption profiles of all samples over the temperature range of 25–40 °C. The reported 95.0 mg/g value (25 °C, 1 bar) was taken from these gravimetric measurements rather than from the static volumetric isotherms. As the adsorption temperature increased, the equilibrium adsorption capacities of all three composites exhibited a clear downward trend, consistent with the exothermic nature of physisorption. Specifically, the capacity of UTSA-16(Co)/PAA-O decreased from 80.3 mg/g to 59.8 mg/g, corresponding to a reduction of 25.5%. That of UTSA-16(Co)/PAA-T dropped from 61.2 mg/g to 47.2 mg/g, with a reduction of 23.0%. Although UTSA-16(Co)/PAA-S showed a larger relative reduction (from 95.0 mg/g to 63.6 mg/g, a reduction of 33.0%), its absolute adsorption capacity at each tested temperature remained markedly higher than that of the other two samples. Overall, these results were in good agreement with the order of MOF mass loading (PAA-S > PAA-O > PAA-T), further confirming that the CO_2_ capture capacity of the composites was mainly determined by the microporous active sites of UTSA-16(Co), while the PAA matrix mainly functioned as a macroscopic shaping medium and structural support.

### 3.4. Effect of CO_2_ Partial Pressure on Adsorption Performance

**Figure 6:**
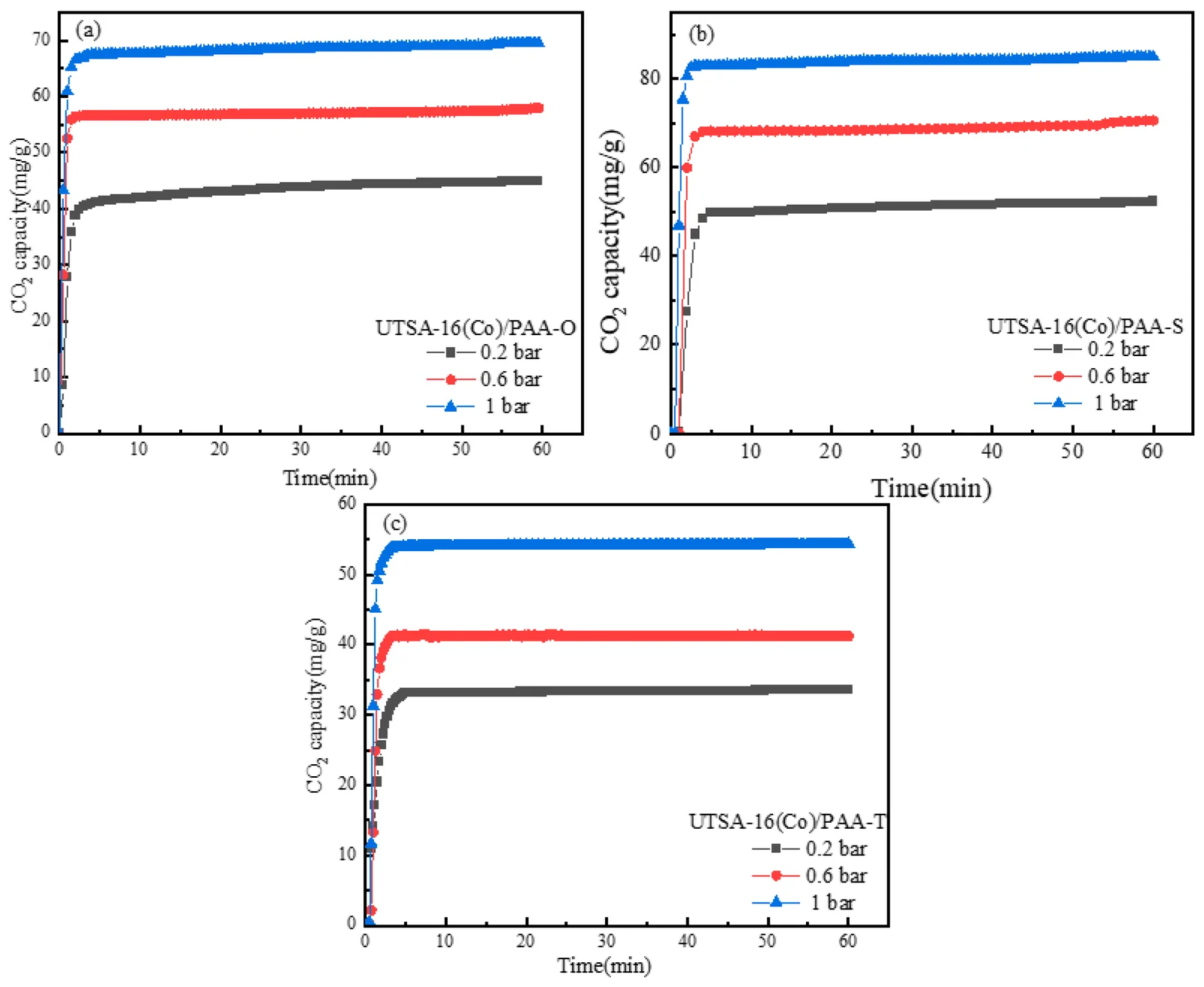
Adsorption curves of (**a**) UTSA-16(Co)/PAA-O, (**b**) UTSA-16(Co)/PAA-S and (**c**) UTSA-16(Co)/PAA-T at different CO_2_ partial pressures.

To further evaluate the influence of CO_2_ partial pressure on adsorption performance, the CO_2_ adsorption isotherms of the three composites were measured at 30 °C within a partial pressure range of 0.2–1 bar (Figure 6, TGA method). The adsorption capacities of UTSA-16(Co)/PAA-O, UTSA-16(Co)/PAA-S, and UTSA-16(Co)/PAA-T increased from 44.9, 52.3, and 33.6 mg/g to 69.5, 84.9, and 54.4 mg/g, respectively, revealing a pronounced positive correlation between adsorption capacity and CO_2_ partial pressure, consistent with the typical behavior of physisorption. More importantly, when the adsorption capacities measured at 0.2 bar were normalized to the unit mass of loaded MOF, the specific adsorption performance per unit mass of active MOF component in the composites even exceeded that of pristine UTSA-16(Co) powder. This enhancement was mainly ascribed to the nanoscale size effect induced by in-situ growth within the confined pore space. As observed by SEM characterization, the in-situ growth process reduced the MOF crystallite size from the micrometer scale to 100–200 nm. This size reduction would greatly diminish intracrystalline diffusion resistance and simultaneously increase the proportion of accessible external surface area for CO_2_ molecules as well as the density of unsaturated surface metal sites, thereby realizing more of the intrinsic adsorption potential of the material under low-partial-pressure conditions.

### 3.5. Adsorption–Desorption Cyclic Stability

**Figure 7:**
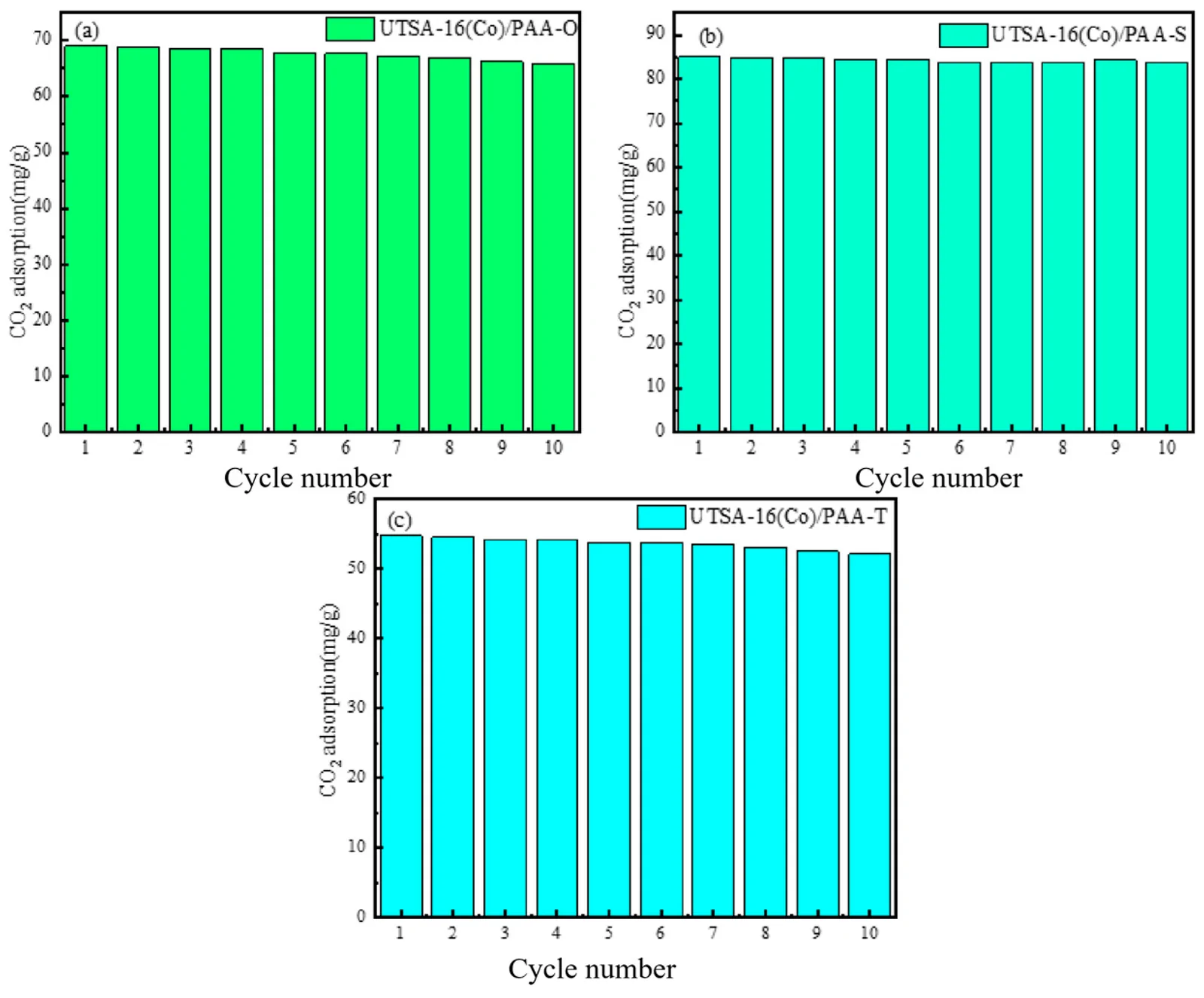
Adsorption capacity of (**a**) UTSA-16(Co)/PAA-O, (**b**) UTSA-16(Co)/PAA-S and (**c**) UTSA-16(Co)/PAA-T during 10 adsorption–desorption cycles.

The cyclic adsorption–desorption stability of adsorbents is a key indicator for evaluating their reusability. To assess this performance, 10 consecutive adsorption–desorption cycle tests were conducted on the three composites at 30 °C (Figure 7). All samples exhibited excellent short-term cyclic stability, among which UTSA-16(Co)/PAA-S exhibited the best overall performance. After 10 cycles, it maintained an adsorption capacity of 83.7 mg/g, corresponding to a capacity retention of 98.6%, with a cumulative capacity decay of only 1.4%. Although the adsorption capacities of all three samples showed a gradual decline as the number of cycles increased, this slight decay was preliminarily attributed to the limited interfacial bonding strength between some MOF crystallites and the inner pore walls of the matrix. Under repeated scouring by the gas flow and cyclic thermal expansion–contraction stresses, local loosening or minor exfoliation of MOF crystallites could occur, leading to partial deactivation of active sites. Nevertheless, the capacity loss of UTSA-16(Co)/PAA-S remained below 2% over 10 cycles, confirming that the MOF–matrix interfacial bonding achieved via the stepwise impregnation method was sufficient to satisfy the requirements of good short-term cyclic stability.

### 3.6. CO_2_ Adsorption Kinetics

(1) Effect of Gas Flow Rate

**Figure 8:**
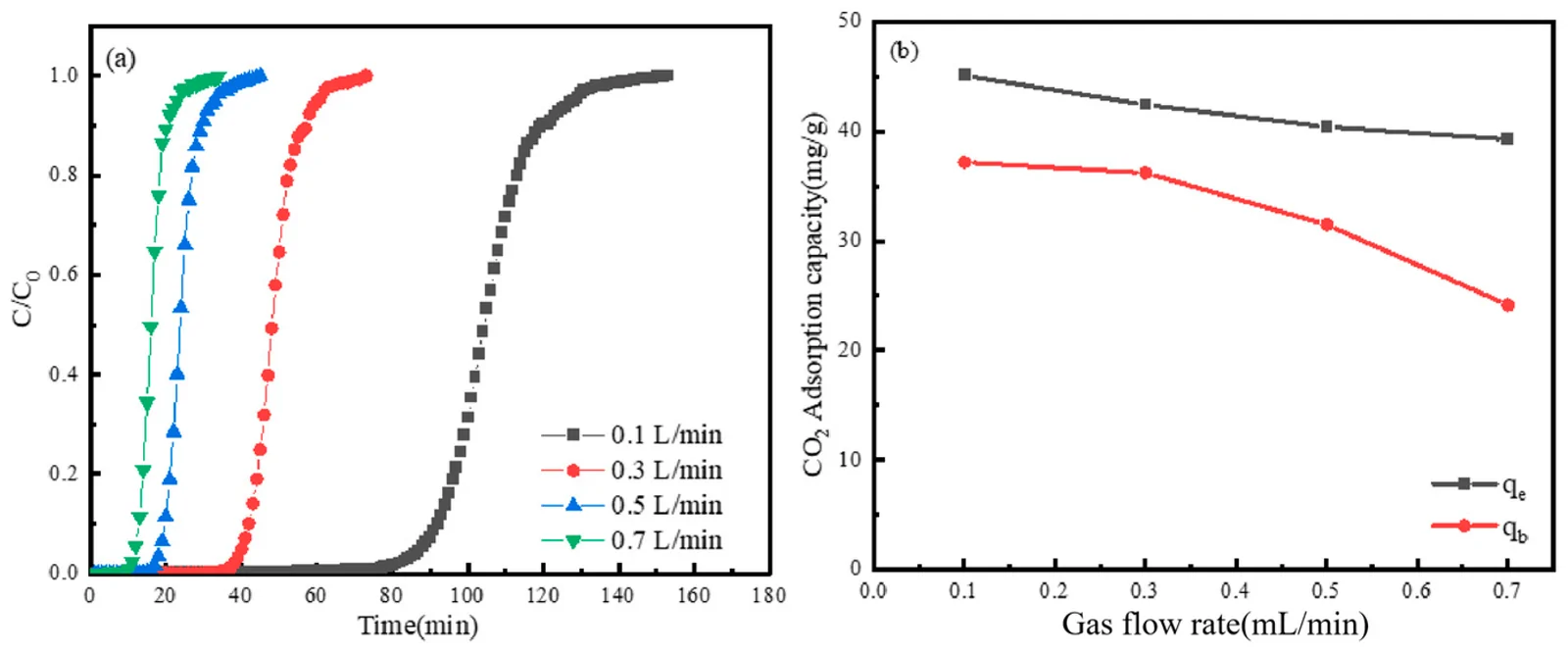
(**a**) CO_2_ adsorption breakthrough curves and (**b**) adsorption capacities at different gas flow rates.

The effect of gas flow rate (0.1–0.7 L/min) on breakthrough performance was investigated at a bed temperature of 25 °C, an inlet CO_2_ concentration of 0.7 vol%, an RH below 1%, and a bed height of 1 cm (Figure 8a). As the flow rate increased, the breakthrough curves exhibited a distinct leftward shift, the breakthrough time decreased significantly, and the mass transfer zone became shorter and steeper. Correspondingly, Figure 8b shows that both the saturated adsorption capacity and the breakthrough adsorption capacity decreased with increasing flow rate. Specifically, when the flow rate increased from 0.1 to 0.7 L/min, the saturated capacity fell from 45 to 39 mg/g, while the breakthrough time decreased drastically from 92 to 13 min. From a kinetic perspective, at a fixed bed height and inlet concentration, a higher flow rate increased the number of CO_2_ molecules entering the bed per unit time and accelerated the rate of gas–solid contact, thereby leading to earlier breakthrough. From a mass transfer perspective, a high flow velocity shortened the gas residence time in the bed, thus causing CO_2_ molecules to be carried out by the gas stream before they could fully diffuse into the inner pore structure of the adsorbent particles and weakening the contribution of intraparticle diffusion. The total number of adsorption sites in the fixed bed remained constant, and the saturated adsorption capacity was dominated by thermodynamic properties, making it far less sensitive to variations in flow rate. To balance the adsorption capacity and breakthrough performance, 0.3 L/min was selected as the optimal flow rate for subsequent experiments.

(2) Effect of Bed Temperature

**Figure 9:**
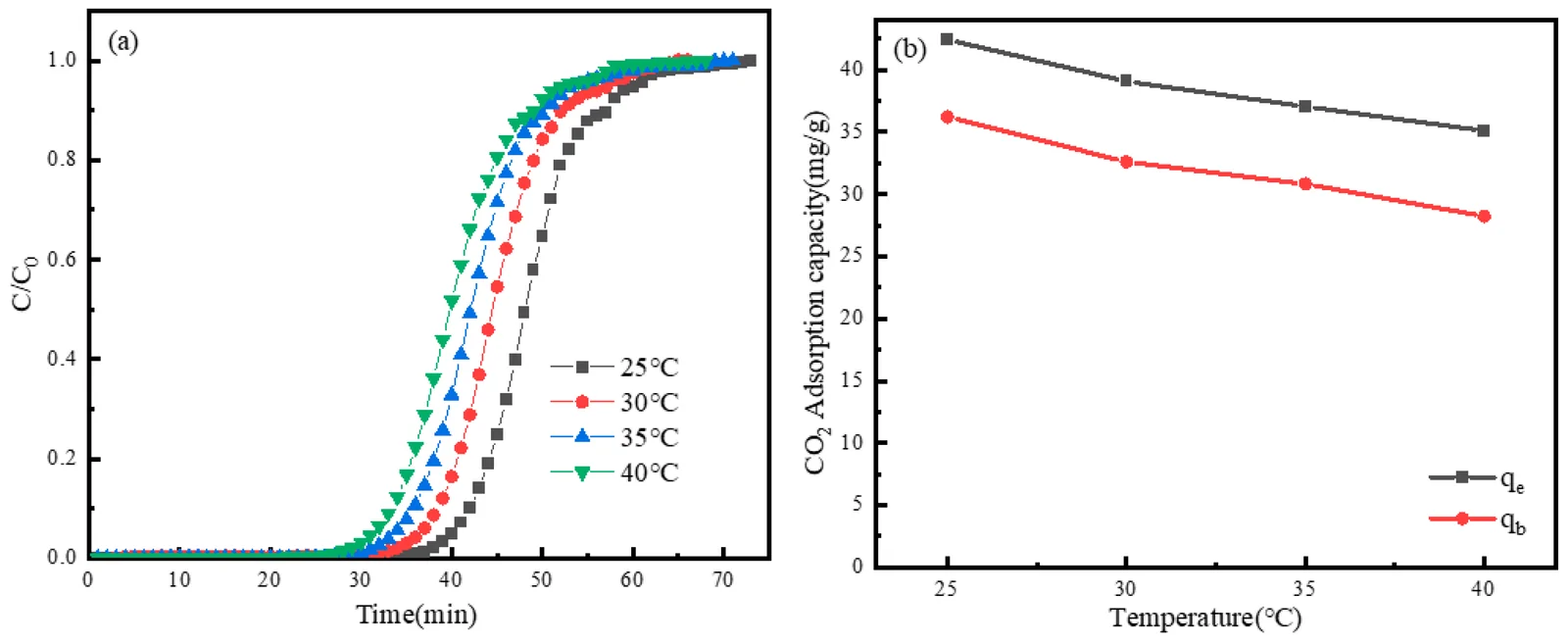
(**a**) CO_2_ adsorption breakthrough curves and (**b**) adsorption capacities at different bed temperatures.

The effect of bed temperature (25–40 °C) was investigated at a fixed flow rate of 0.3 L/min, an inlet CO_2_ concentration of 0.7 vol%, an RH below 1%, and a bed height of 1 cm (Figure 9). The 25–40 °C window was selected because it spans typical indoor/ambient capture conditions and the moderately warm temperatures encountered near post-quench flue gas or enclosed-environment CO_2_ removal, while remaining well below the onset of thermal degradation (>300 °C); it is also the range in which the temperature sensitivity of the predominantly physisorptive uptake is most clearly resolved. As the bed temperature increased, the breakthrough curves shifted markedly leftward. The breakthrough time decreased from 42 min to 33.5 min, and the saturated adsorption capacity dropped from 42.42 mg/g to 35 mg/g, corresponding to a reduction of approximately 17%. These results indicated that temperature exerted a dual effect on the dynamic adsorption process. On the one hand, a higher temperature intensified the thermal motion of gas molecules and increased the effective diffusion coefficient, thereby accelerating the forward advancement of the mass transfer zone and resulting in a steeper breakthrough curve slope and earlier breakthrough. On the other hand, since physisorption was inherently exothermic, elevated temperature was thermodynamically unfavorable for attaining adsorption equilibrium, leading to a decreased equilibrium adsorption capacity. Therefore, in practical engineering applications, a trade-off between the mass transfer rate and saturated adsorption capacity was required to identify the optimal operating temperature.

(3) Effect of Inlet CO_2_ Concentration

**Figure 10:**
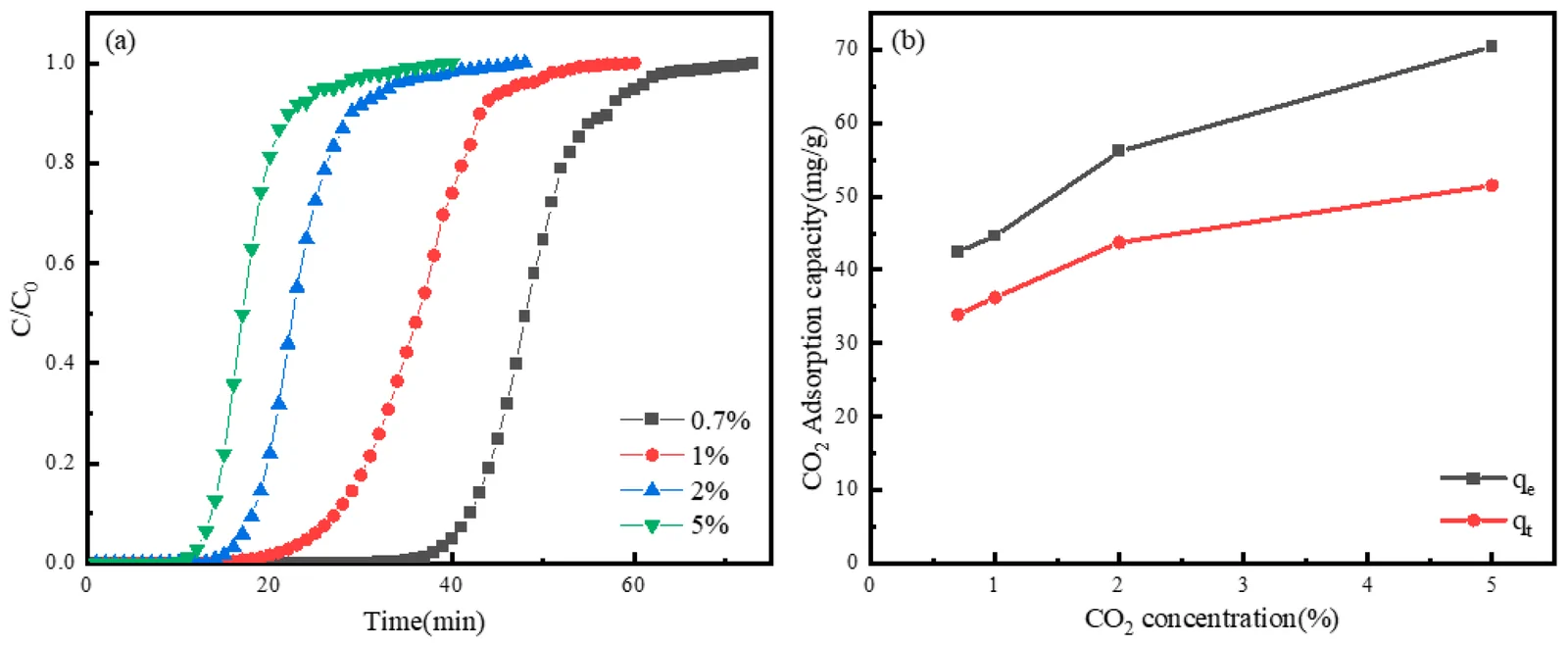
(**a**) CO_2_ adsorption breakthrough curves and (**b**) adsorption capacities at different initial concentrations.

The effect of inlet CO_2_ concentration (0.7–5 vol%) was investigated at a fixed flow rate of 0.3 L/min, a bed temperature of 25 °C, an RH below 1%, and a bed height of 1 cm (Figure 10). As the inlet concentration increased, the breakthrough curves became progressively steeper, and the breakthrough time decreased markedly, specifically from 42 min at 0.7 vol% to 27 min at 1 vol%. Meanwhile, the saturated adsorption capacity increased significantly with increasing inlet concentration, from 42.42 mg/g at 0.7 vol% to 70.14 mg/g at 5 vol%. This phenomenon could be attributed to the elevated mass transfer driving force. On the one hand, a higher inlet concentration increased the concentration gradient between the bulk gas phase and the adsorbent surface, thereby accelerating the diffusion rate and collision frequency of CO_2_ molecules with the adsorption surface. This led to faster occupancy of active adsorption sites and earlier breakthrough. On the other hand, the increased inlet concentration raised the partial pressure of CO_2_ in the gas phase and enhanced the overall mass transfer driving force, allowing more CO_2_ molecules to diffuse into the internal pores of the adsorbent and occupy active sites. This ultimately resulted in a higher saturated adsorption capacity at equilibrium.

(4) Effect of Relative Humidity

**Figure 11:**
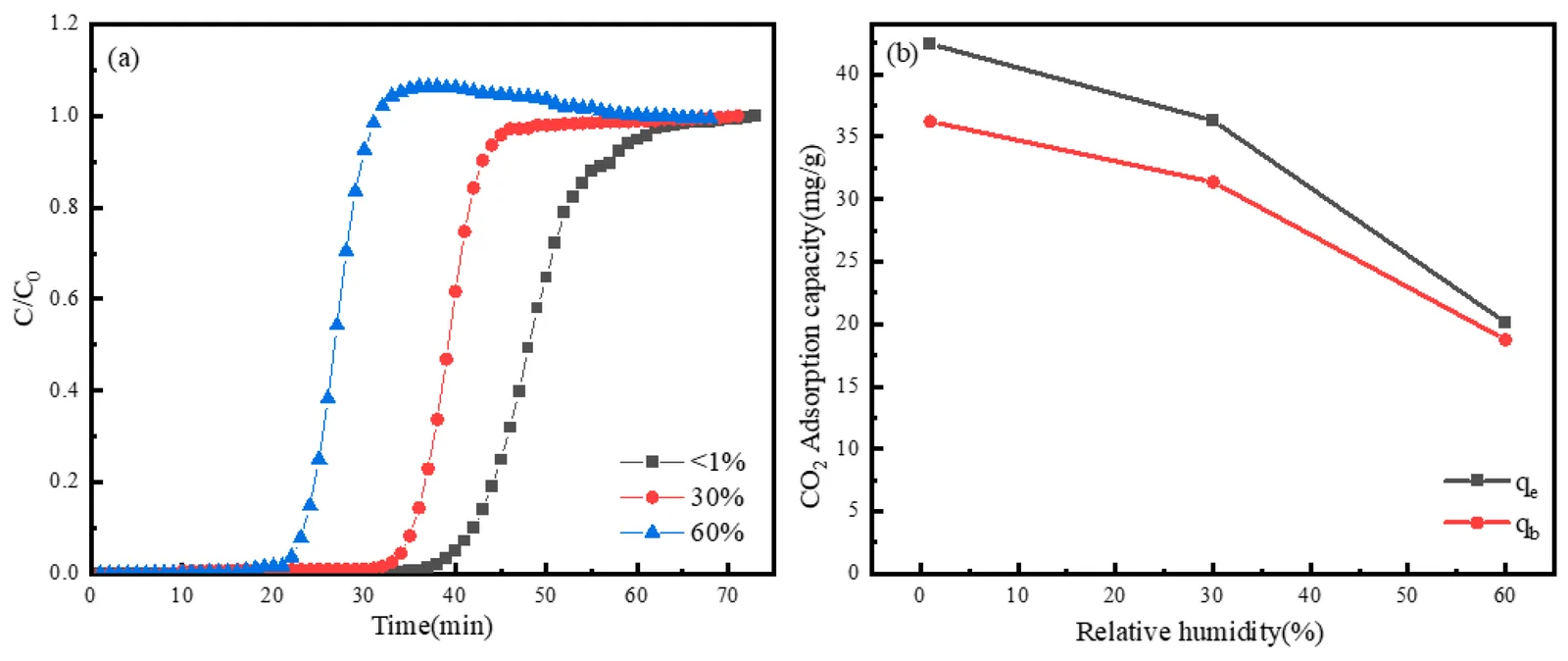
(**a**) CO_2_ adsorption breakthrough curves and (**b**) adsorption capacities at different relative humidities.

Since water vapor was inevitably present in actual flue gas streams, its effect on adsorption performance was evaluated at RH levels below 1%, 30%, and 60% (Figure 11). As RH increased, the breakthrough time shortened progressively, decreasing to 35.5 min at 30% RH and further to 23.5 min at 60% RH. Notably, at 60% RH, a typical roll-up phenomenon emerged on the trailing edge of the breakthrough curve, i.e., the outlet concentration temporarily exceeded the inlet concentration (C/C_0_ > 1). Correspondingly, the saturated adsorption capacity dropped sharply to 20.11 mg/g, corresponding to a 52.6% reduction compared with that under dry conditions. At low RH, the breakthrough curves maintained a typical sigmoidal shape, whereas the roll-up behavior at high RH was consistent with strong competitive adsorption between H_2_O and CO_2_ for adsorption sites. Because water molecules were highly polar, their interactions with the adsorbent surface (e.g., hydrogen bonding and coordination) were much stronger than the van der Waals forces governing CO_2_ physisorption. A plausible explanation was that water molecules gradually displaced pre-adsorbed CO_2_ molecules, causing partial CO_2_ desorption and carrying the displaced CO_2_ out of the bed with the bulk gas stream, which triggered the outlet concentration overshoot. These findings were consistent with previous reports on the detrimental effect of water vapor on CO_2_ adsorption over UTSA-16 and other porous adsorbents [23,24].

(5) Effect of Bed Height

**Figure 12:**
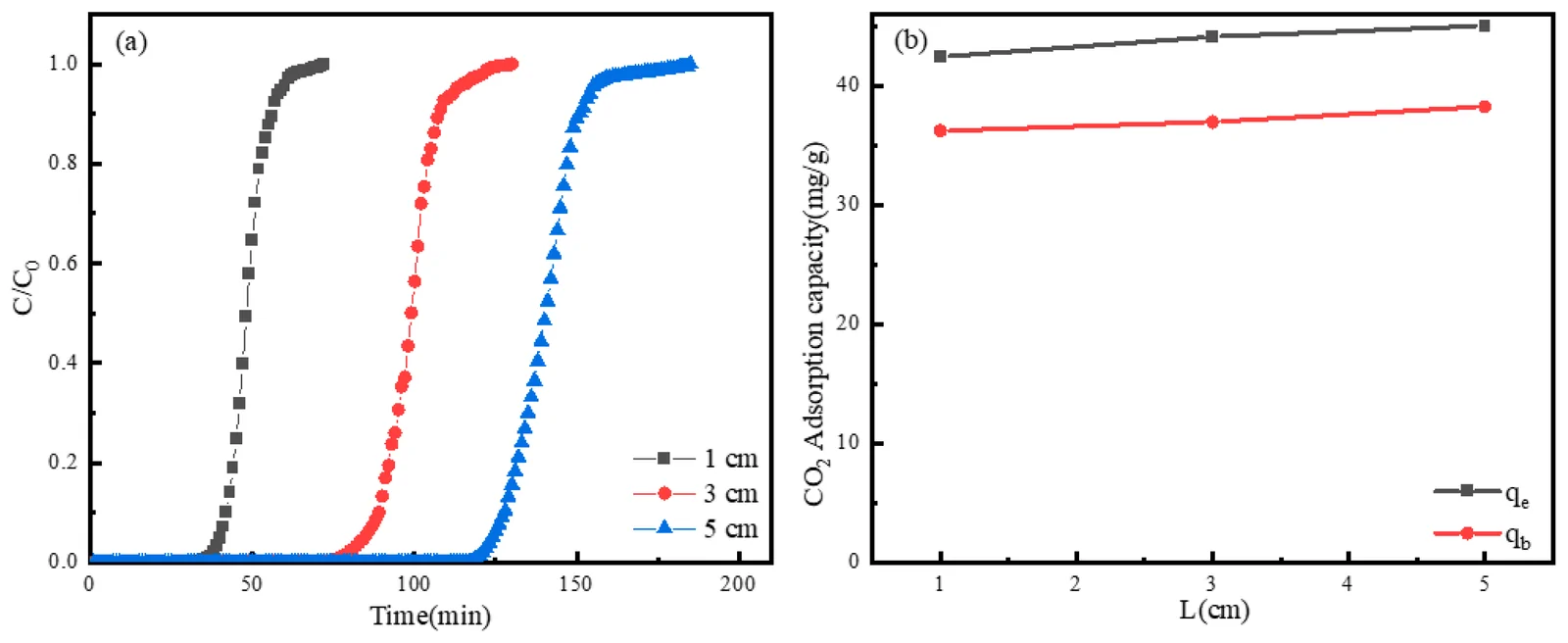
(**a**) CO_2_ adsorption breakthrough curves and (**b**) adsorption capacities at different bed heights.

The effect of bed height (1–5 cm) was investigated at a fixed flow rate of 0.3 L/min, a bed temperature of 25 °C, an inlet CO_2_ concentration of 0.7 vol%, and an RH below 1% (Figure 12). As the bed height increased, the breakthrough curves shifted rightward, and the breakthrough time increased significantly, reaching 128 min at a bed height of 5 cm. Correspondingly, the total saturated adsorption capacity of the whole bed increased with bed height, while the length of the mass transfer zone remained essentially unchanged. A longer bed prolonged the gas residence time and enhanced gas–solid contact. At a fixed flow rate and inlet concentration, the relative contribution of axial convection diminished, allowing radial diffusion and intraparticle mass transfer to proceed more completely. Increasing the bed height under constant operating conditions increased the overall mass transfer coefficient, while the relative length of the mass transfer zone remained largely unchanged. Therefore, bed height mainly governed the breakthrough time and total treatment capacity of the fixed bed, rather than the intrinsic specific saturated adsorption capacity per unit mass of the adsorbent.

(6) Kinetic Fitting of Adsorption–Desorption Processes

The dynamic adsorption capacity curves at temperatures ranging from 25 to 40 °C were calculated via Equation (S3) (Figure S2). All curves exhibited similar two-stage kinetic behavior. The adsorption capacity increased almost linearly during the first 40 min, indicating that this stage was mainly dominated by external film diffusion. Subsequently, the growth rate gradually declined and approached saturation, reflecting the progressive occupation of adsorption sites and the increasing intraparticle diffusion resistance.

To further quantify the adsorption kinetics, the experimental data were fitted using the pseudo-first-order, pseudo-second-order, and Avrami fractional-order kinetic models [25] (Figure S3, Table S2). These models are empirical descriptions of the uptake curves, and a higher coefficient of determination does not, by itself, establish a unique microscopic adsorption mechanism. The pseudo-first-order and pseudo-second-order models yielded correlation coefficients (R^2^) below 0.99, indicating that they could not accurately describe the entire adsorption process. In contrast, the Avrami model achieved R^2^ values above 0.994 at all tested temperatures, exhibiting superior fitting performance relative to the conventional integer-order kinetic models. It is therefore the most suitable empirical model among the three for describing the heterogeneous, multi-stage uptake of CO_2_ onto the composite. The fitted Avrami exponent n varied only weakly with temperature, from 1.58 at 25 °C to 1.65 at 40 °C. An exponent between 1 and 2 indicates that adsorption does not follow a single, integer-order pathway and is consistent with several concurrent processes—external film diffusion, intraparticle diffusion, and occupancy of energetically heterogeneous sites—contributing at different stages; in this empirical context, n reflects the dimensionality/cooperativity of the overall uptake rather than proving a specific nucleation or chemisorption mechanism. Moreover, the Avrami rate constant (kA) increased with rising temperature (0.00419 to 0.00484 min^−1^), which was consistent with the intensified molecular thermal motion and accelerated mass transfer at higher temperatures [26].

### 3.7. CO_2_ Desorption Kinetics

(1) Effect of Purge Gas Flow Rate

**Figure 13:**
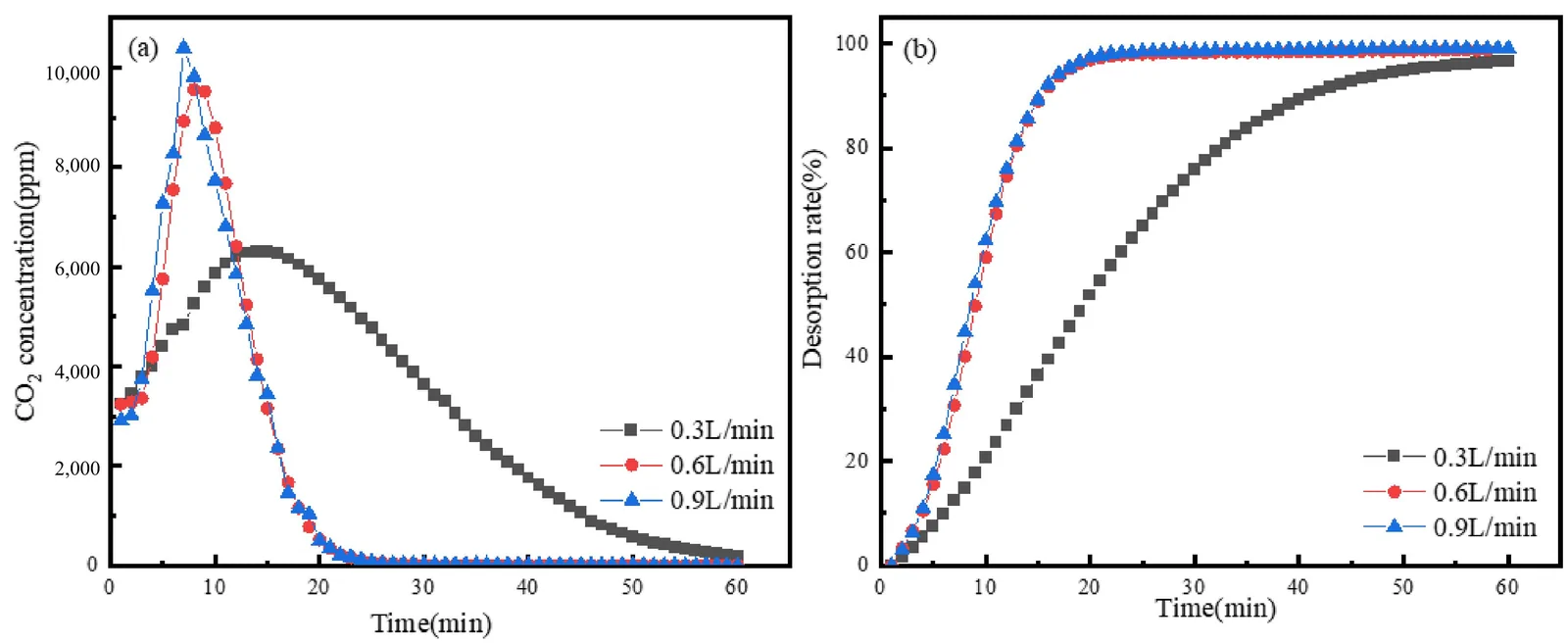
(**a**) CO_2_ desorption curves and (**b**) desorption efficiencies under different purge gas flow rates.

The effect of purge gas flow rate (0.3, 0.6, and 0.9 L/min) on CO_2_ desorption performance was systematically investigated at a desorption temperature of 90 °C after adsorption saturation (Figure 13a). At flow rates of 0.6 and 0.9 L/min, the outlet CO_2_ concentration rose rapidly and reached its peak within the first 10 min. This corresponded to the heating stage, during which the fixed bed was rapidly heated from room temperature to the target temperature and a strong mass transfer driving force was established. Subsequently, the concentration declined gradually during the isothermal desorption stage as the desorbable CO_2_ was gradually depleted, and the CO_2_ concentration dropped to an almost undetectable level by 20 min, indicating complete desorption. In contrast, at a flow rate of 0.3 L/min, the concentration peak was delayed to 15 min, the concentration decay was significantly slower, and complete desorption required 55 min. These results demonstrated that increasing the purge gas flow rate enhanced gas-phase convective mass transfer within the bed and reduced gas film diffusion resistance, thereby accelerating the desorption process and shortening the total desorption time. As shown in Figure 13b, the cumulative desorption capacity and desorption efficiency increased more rapidly at higher purge gas flow rates. However, when the flow rate was increased from 0.6 to 0.9 L/min, the desorption efficiency curves for these two conditions nearly coincided, with both exceeding 99% within 20 min. This indicated that the marginal improvement in desorption efficiency from further increasing the flow rate was very limited. Therefore, to balance desorption efficiency and energy consumption, 0.6 L/min was selected as the optimal purge gas flow rate for the desorption experiments.

(2) Effect of Desorption Temperature

**Figure 14:**
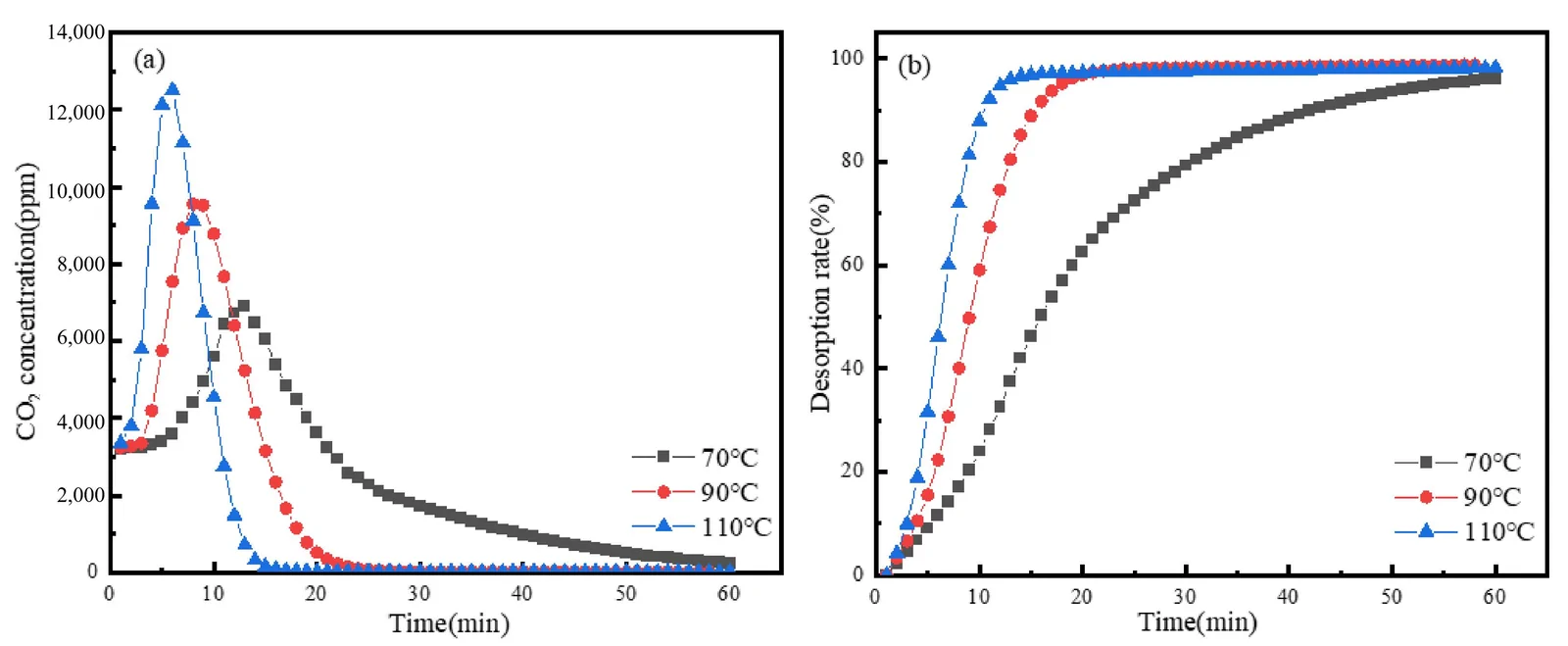
(**a**) CO_2_ desorption curves and (**b**) desorption efficiencies at different desorption temperatures.

The effect of desorption temperature (70, 90, and 110 °C) on desorption performance was further investigated at a fixed purge gas flow rate of 0.6 L/min after adsorption saturation (Figure 14a). As the desorption temperature increased, the peak outlet CO_2_ concentration during desorption rose markedly, reaching 12,500 ppm at 110 °C. The cumulative desorption capacity and desorption efficiency curves (Figure 14b) showed that a desorption efficiency exceeding 99% was achieved in only 15 min at 110 °C, whereas 60 min was required to reach the same level at 70 °C. The elevated temperature reduced the adsorption equilibrium constant and shifted the adsorption–desorption equilibrium toward desorption. In addition, a higher temperature reduced both intraparticle and external film mass transfer resistance, accelerating the diffusion of desorbed CO_2_ from the pore channels to the bulk gas phase. Nevertheless, the performance gain from further increasing the desorption temperature beyond 90 °C was marginal. Although 110 °C reduced the complete desorption time by 5 min relative to 90 °C, excessively high desorption temperatures significantly increased the thermal load and prolonged the cooling period before the next adsorption cycle, thereby reducing the overall cyclic operation efficiency. From the perspective of engineering economy and overall cyclic operation, 90 °C was an appropriate desorption temperature within the laboratory-scale operating window studied.

### 3.8. Reconciliation of Capacities, Comparison with Literature, and Limitations

Three different measurement routes were used in this work, and because they probe the adsorbent under different CO_2_ activities, flow conditions, and definitions of saturation, they necessarily give different numbers; they should not be compared directly without specifying the method. Table S3 reconciles the capacities of the optimal UTSA-16(Co)/PAA-S. The three preparation routes provide a coherent, self-consistent structure–performance trend. As the route changes from secondary growth (T) to one-step (O) to stepwise (S), the Co mass fraction (7.5, 8.13, 13.28%), MOF loading (32, 41, 53 wt%), BET surface area (250.69, 316.97, 407.46 m^2^/g), micropore volume (0.0893, 0.1153, 0.1448 cm^3^/g), and pure-CO_2_ gravimetric uptake at 25 °C (61.2, 80.3, 95.0 mg/g) all increase together. This strong positive correlation supports the view that the stepwise route maximizes the amount of well-dispersed, accessible MOF, and that capacity is governed primarily by MOF-derived micropores; it also explains why the per-unit-mass MOF uptake can exceed that of the powder.

Table S4 positions the composite against previously reported structured UTSA-16/MOF–polymer systems; traditional adsorbents are compared qualitatively in the Introduction. The comparison shows that the present composite is competitive with other MOF/polymer systems at a moderate loading and, importantly, is accompanied by a complete multi-variable fixed-bed and desorption data set that most static-capacity studies do not provide. Its per-MOF normalized efficiency and bead geometry are practical advantages; however, its absolute gravimetric capacity is below that of high-loading PAN beads (~90 wt%) and of the highest-capacity open-site MOFs, so no claim of record capacity is made.

Finally, the present results were obtained at laboratory scale, and several engineering aspects remain to be quantified before scale-up. The compressive strength, attrition/abrasion resistance, particle stability, MOF loss under prolonged flow, and the bed pressure drop were not measured; the shaping advantage is therefore demonstrated at the level of bead geometry and uniform MOF dispersion rather than by quantified engineering performance. The composite is moisture-sensitive (a substantial capacity loss at 60% RH), and quantitative capacity recovery after a humid adsorption–drying cycle, testing beyond ten cycles, and any process/energy/techno-economic evaluation were not examined and are identified as future work. The 90 °C desorption temperature reflects an operating-window optimization within this laboratory study rather than an economic assessment. Finally, each operating condition was measured in a single run, so the reported capacities and kinetic parameters are single determinations. Independent replicate measurements (N ≥ 3) are required to establish experimental repeatability.

## 4. Conclusions

A shaped UTSA-16(Co)/polyacrylate composite adsorbent was fabricated via a stepwise impregnation strategy using PAA-NH_2_ as the porous polymer matrix, which addressed the shaping limitation of pristine MOF powders at the level of bead geometry and uniform dispersion, although quantitative mechanical and engineering performance remains to be evaluated. The stepwise impregnation method enabled uniform nucleation and growth of MOF crystallites within pore channels via spatial confinement, achieving a high MOF loading of 53 wt%, significantly higher than those of the one-step impregnation (41 wt%) and secondary growth (32 wt%) methods. The composite possessed a hierarchical micro-/mesoporous structure. The polymer matrix provided good formability, and the confined pore environment elevated the per-unit-mass MOF adsorption efficiency at 0.2 bar above that of pristine MOF powder. The material delivered a pure-CO_2_ gravimetric uptake of 95.0 mg/g at 25 °C and retained 98.6% of its initial capacity after 10 adsorption–desorption cycles, evidencing good short-term cyclic stability. Systematic fixed-bed breakthrough experiments elucidated the quantitative regulation mechanism of multiple operating parameters on dynamic adsorption behavior. Breakthrough time decreased with increasing gas flow rate, bed temperature, inlet CO_2_ concentration, and RH but increased significantly with increasing bed height. Among the three empirical models, the Avrami fractional-order model (R^2^ ≥ 0.994) exhibited higher fitting accuracy than the pseudo-first-order and pseudo-second-order kinetic models. For thermal purge desorption, the desorption efficiency showed a positive correlation with purge gas flow rate and desorption temperature. A high desorption efficiency (>99%) was achieved within 20 min under optimal conditions (90 °C, 0.6 L/min purge gas). This work provides systematic kinetic data and operational references for the optimization of MOF–polymer composite adsorbents in laboratory-scale fixed beds, laying a foundation for subsequent pilot-scale trials.

## Data Availability

The original contributions presented in this study are included in the article. Further inquiries can be directed to the corresponding authors.

## Supplementary Materials

The following are available online at https://www.mdpi.com/article/10.3390/nano16211341/s1, Figure S1: Adsorption curves of (**a**) UTSA-16(Co)/PAA-S-P6, (**b**) UTSA-16(Co)/PAA-S-P18 and (**c**) UTSA-16(Co)/PAA-S-T composites at different gas flow rates. Figure S2: DynamicCO_2_ adsorption curves of UTSA-16(Co)/PAA-S at different temperatures. Figure S3: Fitting curves of three kinetic models for CO_2_ adsorption at (**a**) 25 °C, (**b**) 30 °C, (**c**) 35 °C and (**d**)40 °C. Table S1: BET specific surface areas, average pore sizes and micropore volumes of UTSA-16(Co), PAA-NH_2_, UTSA-16(Co)/PAA-O, UTSA-16(Co)/PAA-S and UTSA-16(Co)/PAA-T. Table S2: Kinetic fitting parameters for CO_2_ adsorption at different temperatures. Table S3: CO_2_ capacities of the optimal UTSA-16(Co)/PAA-S obtained by the three measurement routes. Table S4: Comparison of representative adsorbents and structured MOF/polymer systems.
