# Supplementary material for: Comprehensive Adsorption–Desorption Kinetics of Shaped UTSA-16(Co)/Polyacrylate Composites for Fixed-Bed CO2 Capture
Source: Nanomaterials (Basel). 2026 Oct 22;16(21):1341. doi: 10.3390/nano16211341 (PMC13764714; doi:10.3390/nano16211341)
Supplement: Supplementary file 1 [file nanomaterials-16-01341-s001.zip › nanomaterials-4565089-supplementary.pdf]

Supplementary Materials

Comprehensive Adsorption–Desorption Kinetics of Shaped UTSA-16(Co)/Polyacrylate Composites for Fixed-Bed CO_2 Capture

Xiangmei Zhang [1,2], Chunlai Wang [1,*], Zhejiaji Zhu [1], Wanyue Zang [1,3], Yuan Sun [1] and Li Lv [1,*]

[1] State Key Laboratory of Chemistry for NBC Hazards Protection, Beijing 100191, China
[2] College of Chemical Engineering, Beijing University of Chemical Technology, Beijing 100029, China
[3] Tianjin Key Laboratory of Green Chemical Technology and Process Engineering, Tiangong University, Tianjin 300387, China
* Correspondence: wangchunlai@sklnbcpc.cn (C.W.); lvli@sklnbcpc.cn (L.L.)

Table S1. BET specific surface areas, average pore sizes and micropore volumes of UTSA-16(Co), PAA-NH_2, UTSA-16(Co)/PAA-O, UTSA-16(Co)/PAA-S and UTSA-16(Co)/PAA-T.

Sample	S_{BET} ($m^2 \cdot g^{-1}$)	S_{micro} ($m^2 \cdot g^{-1}$)	V_{micro} ($cm^3 \cdot g^{-1}$)	Average Pore Diameter (nm)
UTSA-16(Co)	839.14	831.25	0.3052	1.44
PAA-NH_2	35.26	-	-	26.42
UTSA-16(Co)/PAA-O	316.97	296.72	0.1153	2.06
UTSA-16(Co)/PAA-S	407.46	387.13	0.1448	2.27
UTSA-16(Co)/PAA-T	250.69	233.38	0.0893	2.53

Langmuir and Freundlich Adsorption Isotherm Models

The experimental isotherm data were fitted with the Langmuir and Freundlich models. The Langmuir model is applicable to type I adsorption isotherms of microporous materials, and its mathematical expression is shown in **Equation (S1)**:

$$q_e = \frac{q_m \cdot b \cdot P}{1 + b \cdot P} \quad \text{(S1)}$$

where q_e is the equilibrium adsorption capacity (mg/g); q_m is the monolayer saturated adsorption capacity (mg/g); P is the equilibrium partial pressure of CO_2 (Pa); and b is the Langmuir adsorption equilibrium constant.

The Freundlich model is a semi-empirical adsorption isotherm model applicable to multilayer adsorption on heterogeneous adsorbent surfaces. Its expression is given by **Equation (S2)**:

$$q_e = K_F \cdot P^{\frac{1}{n}} \quad \text{(S2)}$$

where q_e is the equilibrium adsorption capacity (mg/g); K_F is the Freundlich constant, which is related to the adsorption capacity; P is the equilibrium partial pressure of CO_2 (Pa); and n is the Freundlich exponent, with 1/n reflecting the adsorption intensity and the surface heterogeneity.

Dynamic Saturated Adsorption Capacity

Breakthrough curves were plotted with the normalized concentration C_t/C_0 on the y-axis and time t on the x-axis. The dynamic saturated adsorption capacity is calculated via numerical integration, as expressed in **Equation (S3)**:

$$q_e = \frac{F \times C_0 \times M \times 10^{-6}}{m \times V_m} \times (t_s - \int_0^{t_s} \frac{C_t}{C_0} dt) \quad \text{(S3)}$$

where q_e is the dynamic saturated adsorption capacity (mg/g); M is the molar mass of CO_2 (g/mol); F is the gas flow rate (mL/min); C_0 and C_t are the inlet and outlet CO_2 concentrations (ppm), respectively; t_s is the saturation time (min); m is the mass of the adsorbent (g); and V_m is the molar volume of gas (L/mol).

Representative Fixed-Bed Capacity Calculation

To improve reproducibility, the following worked example applies Equation (S3) to the run at 25 °C, 0.7 vol% CO_2 (C_0 = 7000 ppm), F = 0.3 L/min (300 mL/min), a bed inner diameter of 20 mm, a bed height of 1 cm, and a representative adsorbent mass of m = 1.5 g. Numerical integration (Simpson's rule at 1 min intervals) of the archived breakthrough curve from t = 0 to the saturation time ts gives $\int(C_0 - C_t)\,dt \approx 1.18 \times 10^5$ ppm·min. Substituting M = 44.01 g/mol and Vm = 24.45 L/mol yields

$q_e = (44.01 \times 300 \times 1.18\times10^5 \times 10^{-6})/(24.45 \times 1.5) \approx 42.4$ mg/g

in agreement with the reported 42.42 mg/g. The breakthrough time is read at C_t/C_0 = 0.05, and the saturation time at C_t/C_0 = 1.0 is maintained for 10 min. Applying the same procedure to the other conditions gives the capacities reported in Figures 8–12.

Adsorption Kinetic Models

Nonlinear regression fitting of the CO_2 adsorption data for UTSA-16(Co)/PAA-S was performed using the pseudo-first-order, pseudo-second-order, and Avrami kinetic models, with their mathematical expressions shown in **Equations (S4) to (S6)**:

Pseudo-first-order kinetic model:

$$q_t = q_e(1 - exp(-k_1 t)) \quad \text{(S4)}$$

Pseudo-second-order kinetic model:

$$q_t = \frac{k_2 q_e^2 t}{1 + k_2 q_e t} \quad \text{(S5)}$$

Avrami kinetic model:

$$q_t = q_e(1 - exp(-(k_A t)^n)) \quad \text{(S6)}$$

where q_e is the equilibrium adsorption capacity (mg/g); q_t is the adsorption capacity at time t (mg/g); t is the adsorption time (min); k_1 is the pseudo-first-order rate constant (min^{-1}); k_2 is the pseudo-second-order rate constant ($g \cdot mg^{-1} \cdot min^{-1}$); k_A is the Avrami rate constant (min^{-1}); and n is the fractional order exponent.

Desorption Capacity and Desorption Efficiency

The CO_2 desorption capacity per unit mass of adsorbent was obtained by integrating the desorption curve, as shown in **Equation (S7)**:

$$q_d = \frac{F \cdot M \cdot 10^{-6}}{m \cdot V_m} \times \int_0^t C_d \mathrm{d}t \quad \text{(S7)}$$

where q_d is the CO_2 desorption capacity (mg/g); F is the purge gas flow rate (mL/min); and C_d is the outlet CO_2 concentration during the desorption process (ppm); M, V_m, and m are the molar mass of CO_2 (g/mol), the molar gas volume (L/mol), and the adsorbent mass (g), respectively.

The CO_2 desorption efficiency was calculated by **Equation (S8)**:

$$\eta = \frac{q_d}{q_e} \times 100\% \quad \text{(S8)}$$

where η is the desorption efficiency (%); q_e is the saturated CO_2 adsorption capacity (mg/g); and q_d is the desorption capacity as defined in Equation (S7).

MOF Mass Loading

The MOF mass loading was calculated by **Equation (S9)**:

$$w\% = \frac{m_2 - m_1}{m_2} \times 100 \quad \text{(S9)}$$

where w is the loading ratio; m_2 is the mass of the composite (g); and m_1 is the mass of the pristine polymer matrix (g).

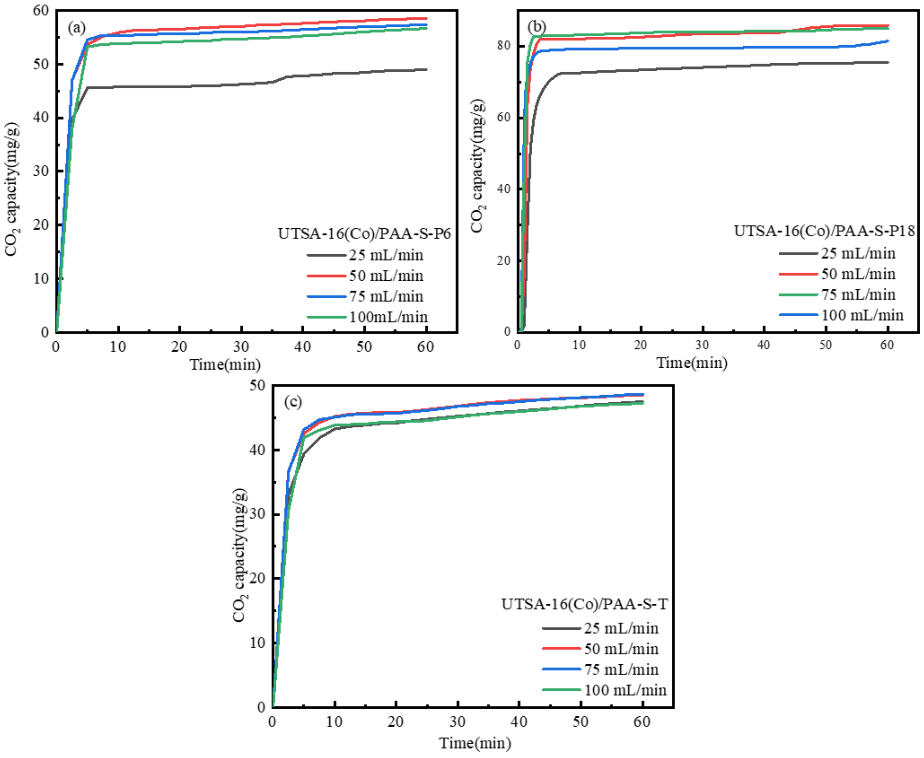

Figure S1. Adsorption curves of (**a**) UTSA-16(Co)/PAA-S-P6, (**b**) UTSA-16(Co)/PAA-S-P18 and (**c**) UTSA-16(Co)/PAA-S-T composites at different gas flow rates.

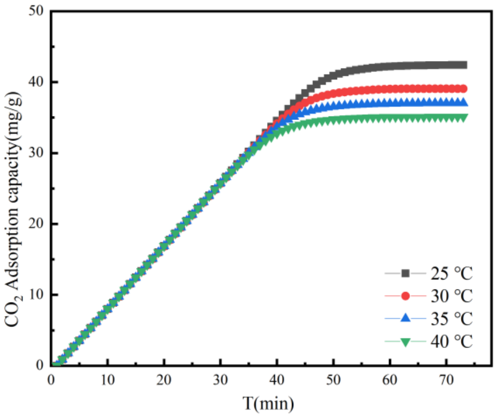

Figure S2. Dynamic CO_2 adsorption curves of UTSA-16(Co)/PAA-S at different temperatures.

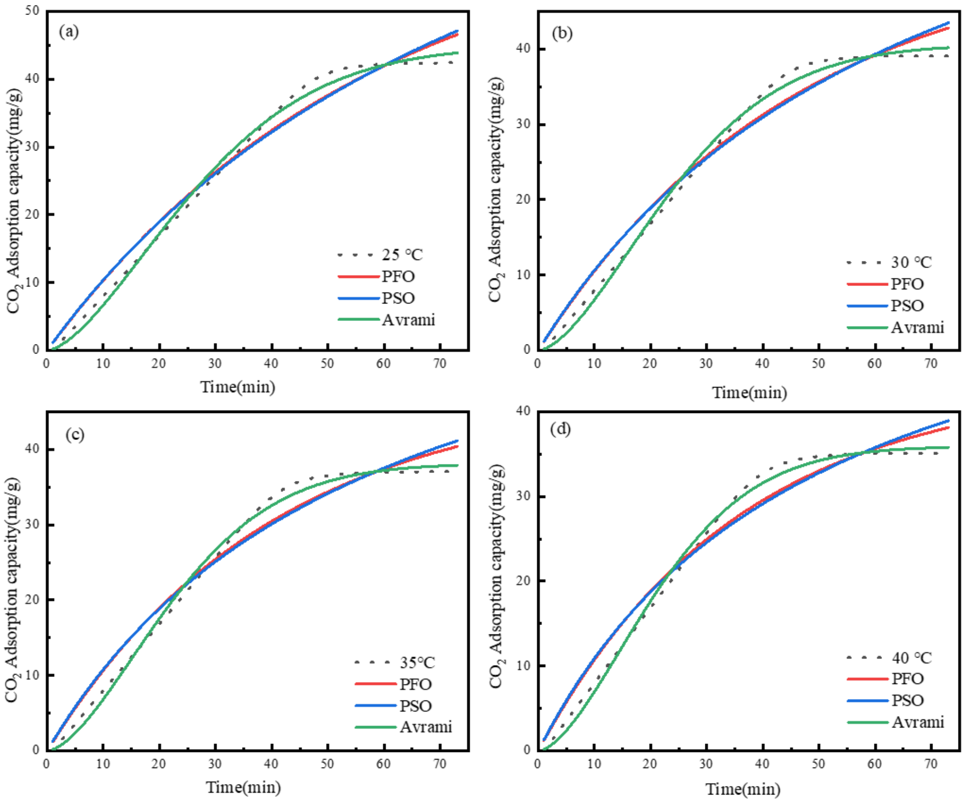

Figure S3. Fitting curves of three kinetic models for CO_2 adsorption at (**a**) 25 °C, (**b**) 30 °C, (**c**) 35 °C and (**d**) 40 °C.

Table S2. Kinetic fitting parameters for CO_2 adsorption at different temperatures.

Model	Parameter	25 °C	30 °C	35 °C	40 °C
Pseudo-first-order	q_e ($mg \cdot g^{-1}$)	64.84	53.71	48.06	43.66
	k_1 (min^{-1})	0.017	0.022	0.025	0.028
	R^2	0.976	0.971	0.969	0.967
Pseudo-second-order	q_e ($mg \cdot g^{-1}$)	107.41	85.28	74.16	65.69
	k_2 ($10^{-4} \cdot g \cdot mg^{-1} \cdot min^{-1}$)	0.99	1.67	2.31	3.04
	R^2	0.973	0.966	0.962	0.958
Avrami	q_e ($mg \cdot g^{-1}$)	44.89	40.59	38.11	35.90
	k_A (min^{-1})	0.00419	0.00429	0.00453	0.00484
	n	1.58	1.62	1.64	1.65
	R^2	0.994	0.994	0.995	0.996

Table S3. CO_2 capacities of the optimal UTSA-16(Co)/PAA-S obtained by the three measurement routes.

Method	Conditions	Gas Feed	Capacity	Figure
Static volumetric (Autosorb IQ)	0 °C, 0–1 bar	pure CO_2	Type-I isotherm; physisorption	
Static volumetric (Autosorb IQ)	25 °C, 0–1 bar	pure CO_2	Type-I isotherm; physisorption	
Gravimetric TGA (pure CO_2)	25 °C, 1 bar	pure CO_2	95.0 mg/g	Fig. 5
Gravimetric TGA (pure CO_2)	25→40 °C	pure CO_2	95.0→63.6 mg/g	Fig. 5
Gravimetric TGA (pure CO_2)	30 °C, 0.2–1 bar	pure CO_2	52.3→84.9 mg/g	Fig. 6
Gravimetric TGA, cycling	30 °C, 10 cycles	pure CO_2	84.9→83.7 mg/g (98.6%)	Fig. 7
Dynamic fixed-bed	25 °C, 0.7 vol%, 0.3 L/min	0.7% CO_2/air	42.42 mg/g	Fig. 9
Dynamic fixed-bed	25 °C, 5 vol%, 0.3 L/min	5% CO_2/air	70.14 mg/g	Fig. 10
Dynamic fixed-bed	25 °C, 60% RH, 0.7 vol%	humid	20.11 mg/g (−52.6%)	Fig. 11

Table S4. Comparison of representative adsorbents and structured MOF/polymer systems.

Adsorbent/ System	Shaping/ Polymer	MOF Loading	CO_2 Capacity (25 °C, 1 Bar, Unless Noted)	Ref.
UTSA-16 powder	—	100%	~3.5 mmol/g (154 mg/g)	[7]
Electrospun PAN/UTSA-16 fiber	fiber (PAN)	varied	retains >75% of pristine UTSA-16 CO_2 uptake	[8]
UTSA-16/3D-printed kaolin monolith	monolith (clay)	proportional	3.1 mmol/g (powder 3.5)	[9]
NbOFFIVE-1-Ni/polyacrylate bead	bead (polyacrylate)	45.8 wt%	1.44 mmol/g at 400 ppm, 298 K (DAC)	[10]
SIFSIX-3-Ni/PAN bead	bead (PAN)	varied	0.57–1.74 mmol/g at 1 bar	[11]
MOF/PAN phase-inversion bead	bead (PAN)	up to ~90 wt%	retains MOF uptake (UiO-66/ZIF-8)	[12]
UTSA-16(Co)/PAA-S (this work)	porous bead ($PAA-NH_2$)	53 wt%	95.0 mg/g (2.16 mmol/g), pure CO_2; 70.14 mg/g at 5 vol%	this work
